# Targeting Mesenchymal-Epidermal Transition (MET) Aberrations in Non-Small Cell Lung Cancer: Current Challenges and Therapeutic Advances

**DOI:** 10.3390/cancers18020207

**Published:** 2026-01-08

**Authors:** Fahua Deng, Weijie Ma, Sixi Wei

**Affiliations:** 1 Center for Clinical Laboratories, Hospital Affiliated to Guizhou Medical University, Guiyang 550004, China; 2 Department of Pathology and Laboratory Medicine, Dartmouth Hitchcock Medical Center, Geisel School of Medicine at Dartmouth, Lebanon, NH 03756, USA

**Keywords:** MET genetic alterations, NSCLC, TKI resistance, diagnostics, combination therapy

## Abstract

The mesenchymal–epithelial transition (MET) receptor is a tyrosine kinase activated by its sole known ligand, hepatocyte growth factor (HGF). MET signaling regulates key cellular processes, including proliferation, survival, migration, motility, and angiogenesis. Dysregulation and hyperactivation of this pathway are implicated in multiple malignancies, including lung, breast, colorectal, and gastrointestinal cancers. In non–small cell lung cancer (NSCLC), aberrant activation of the *MET* proto-oncogene contributes to 1% of known oncogenic drivers and is associated with poor clinical outcomes. Several mechanisms can induce MET hyperactivation, including *MET* gene amplification, transcriptional upregulation of *MET* or *HGF*, *MET* fusion genes, and *MET* exon 14 skipping mutations. Furthermore, MET pathway activation represents a frequent mechanism of acquired resistance to EGFR- and ALK-targeted tyrosine kinase inhibitors (TKIs) in EGFR- and ALK-driven NSCLCs. Although MET has long been recognized as a promising therapeutic target in NSCLC, the clinical efficacy of MET-targeted therapies has historically lagged behind that of EGFR and ALK inhibitors. Encouragingly, several MET TKIs such as capmatinib, tepotinib, and savolitinib have been approved for the treatment of *MET* exon 14 skipping mutations. They have also demonstrated potential in overcoming MET-driven resistance to EGFR TKIs or ALK TKIs. On 14 May 2025, the U.S. Food and Drug Administration granted accelerated approval to telisotuzumab vedotin-tllv for adult patients with locally advanced or metastatic non-squamous NSCLC whose tumors exhibit high c-Met protein overexpression and who have already received prior systemic therapy. In this review, we summarize the structure and physiological role of the MET receptor, the molecular mechanisms underlying aberrant MET activation, its contribution to acquired resistance against targeted therapies, and emerging strategies for effectively targeting MET alterations in NSCLC.

## Simple Summary

Non-small cell lung cancer (NSCLC) represents the most prevalent form of lung cancer. Abnormal activation of mesenchymal transition (MET) proto-oncogenes serves as a key oncogenic driver in NSCLC and is correlated with poor clinical outcomes. The activation of the MET pathway is not only a carcinogenic factor for NSCLC but also a highly potential therapeutic target. Such abnormal MET activation may occur through *MET* gene mutations, *MET* gene amplification or transcriptional upregulation without amplification. Although MET tyrosine kinase inhibitors (TKIs) are currently the most widely employed class of MET-targeted agents, their clinical benefits remain limited and insufficient to fully meet clinical needs. In recent years, monoclonal antibodies directed against specific MET epitopes have demonstrated encouraging clinical efficacy in various trials. Furthermore, combination therapeutic strategies—especially with EGFR inhibitors—have demonstrated greater clinical potential compared to monotherapy by overcoming drug resistance and enhancing efficacy. Ongoing research in this field is essential to fully integrate MET-targeted therapies into routine clinical practice and expand treatment options for patients with NSCLC. This review comprehensively summarizes the structure and physiological functions of the MET receptor, the molecular mechanisms underlying aberrant MET activation, its role in acquired resistance to targeted therapies, and emerging strategies for effectively targeting MET alterations in NSCLC.

## 1. Introduction

The mesenchymal-epidermal transition factor receptor (MET), also known as hepatocyte growth factor receptor (HGFR), was originally identified as the protein product of a transforming gene in a chemically transformed osteosarcoma cell line [1]. In 1991, MET was discovered to be the receptor for hepatocyte growth factor (HGF), also called scatter factor (SF), a protein previously shown to promote hepatocyte growth in culture [2]. In the following decade, *MET* was found to be a potent oncogene with intracellular tyrosine kinase activity in multiple cancer types, including non-small cell lung cancer (NSCLC). In some NSCLCs, the MET pathway is thought to be the primary driving mechanism and sometimes acts as a cellular proto-oncogene. Although MET has been recognized as a promising therapeutic target, MET-targeted drug therapies have thus far achieved only limited success.

**Figure 1:**
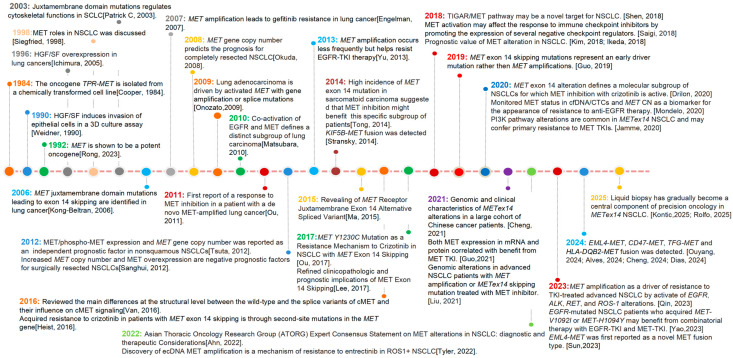
Milestones of discovery for MET alterations in lung cancers [1,2,3,4,5,6,7,8,9,10,11,12,13,14,15,16,17,18,19,20,21,22,23,24,25,26,27,28,29,30,31,32,33,34,35,36,37,38,39,40,41,42,43,44].

**Figure 2:**
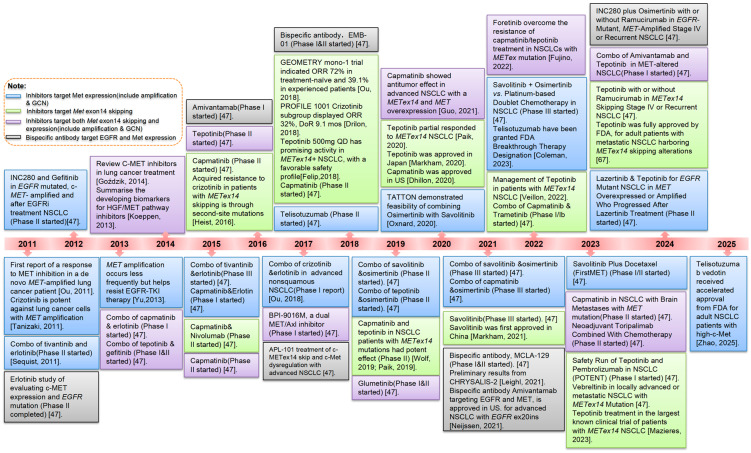
Milestones of treatment for MET alterations in lung cancers [12,15,20,45,46,47,48,49,50,51,52,53,54,55,56,57,58,59,60,61,62,63,64,65,66,67,68].

Figure 1 summarizes the timeline of MET research and the major development milestones of MET-targeted drug therapies in lung cancer. In this review, we summarize the current knowledge on the pathogenesis of MET alterations, methods to detect MET alterations, rationale and therapeutic strategies for targeting MET alterations in patients with advanced NSCLC. Figure 2 reviews milestones of treatment and clinical trial for MET alterations in lung cancers.

## 2. Structure and Normal Function of HGF/MET

Normal MET signaling is active during embryonic development, where it plays roles in gastrulation, angiogenesis, mesenchymal cell migration, bone and muscle formation, and organogenesis [69]. In adults, MET signaling is restricted to functions in wound repair, organ regeneration, and hematopoietic cell differentiation [70]. MET is normally expressed by cells of epithelial-endothelial origin, while its ligand HGF is secreted by mesenchymal cells [71]. The *MET* gene, located on chromosome 7q21-31, has a length of 110 kb and includes 21 exons. It encodes a transmembrane receptor belonging to the RTK superfamily and the HGF receptor family.

**Figure 3:**
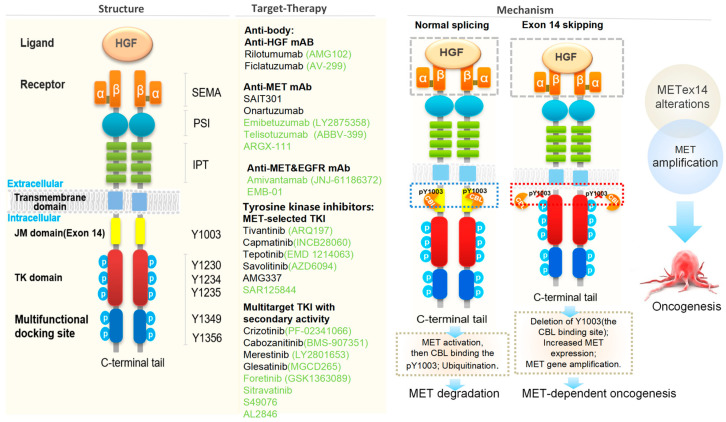
Schematic structure of c-MET protein and the strategies for targeting the HGF-MET signaling pathway [16,71,73,76,77,78,79,80].

The MET receptor is assembled from an α-subunit and a β-subunit derived from the cleavage of a single-chain precursor; in the mature receptor, the extracellular α-subunit is linked to the transmembrane β-subunit by a disulfide bond. The extracellular portion of MET, responsible for binding HGF, is composed of a semaphorin homology (SEMA) domain, a cysteine-rich plexin-semaphorin-integrins (PSI) domain, and four immunoglobulins-plexins-transcription factors (IPT) repeats of immunoglobulin (Ig)-like modules [72,73]. The intracellular portion of MET is composed of a juxtamembrane (JM) domain, a tyrosine kinase (TK) domain, and a C-terminal region with a multifunctional docking site, which are collectively responsible for signal transduction and biological responses [74]. The juxtamembrane domain plays a role in the negative regulation of MET activity and receptor degradation, while the tyrosine kinase domain contains the ATP-binding site and is responsible for receptor autophosphorylation [10]. A multifunctional docking site in the C-terminal region interacts with multiple substrates, including growth factor receptor-bound protein 2 (Grb2), Grb2-associated binder (GAB1) Src homology 2 domain containing (SHC), and Steroid receptor coactivator 2(Scr2), allowing for phosphorylation of signal transducers in downstream pathways [72,75] (Figure 3).

MET is activated when its ligand HGF binds to the MET receptor, inducing homodimerization and phosphorylation of intracellular tyrosine residues [81]. Also known as scatter factor (SF), HGF is primarily secreted by cancer-associated fibroblasts (CAFs) and represents the only known mammalian agonistic natural ligand with high affinity for MET. As the core stromal cells of the tumor microenvironment, CAFs can be activated by cytokines (e.g., TGF-β and IL-6) secreted by tumor cells with *KRas* mutations. Once activated, CAFs secrete substantial amounts of HGF, which subsequently binds to MET on the tumor cell surface via paracrine signaling to activate the MET pathway. HGF is initially secreted as an inactive single-chain precursor, converted by proteases into a biologically active mature heterodimer composed of an α-chain and a β-chain. Upon binding to HGF, the MET receptor undergoes autophosphorylation of the Y1234 and Y1235 tyrosine residues in the kinase domain. Subsequently, tyrosine residues in the docking site (Y1349 and Y1356) become phosphorylated, allowing for downstream signaling through pathways variously involved in cellular proliferation, survival, migration, motility, invasion, angiogenesis, and the epithelial-to-mesenchymal transition. Downstream pathways activated by MET signaling include the phosphoinositide 3-kinase/Akt pathway (PI3K/AKT), signal transducer and activator of transcription 3 (STAT3), the Wnt/β-catenin pathway, and the Erk/mitogen-activated protein kinase cascade (ERK/MAPK) [76,82].

HGF/MET axis-activated drug resistance is dependent on the synergistic interplay of the tumor microenvironment and often overlaps with immunosuppressive states to exacerbate treatment resistance. Studies have shown that in the co-culture model of *KRas* G12C-mutated lung cancer cell lines (H358, A549) and CAFs, the HGF secreted by CAFs can markedly attenuated the antitumor efficacy of sotorasib, while the combined use of camatinib can effectively block the HGF/MET axis and restore the sensitivity of tumor cells to sotoracib [83]. Furthermore, targeted inhibition of upstream signaling pathways (such as the TGF-β pathway) regulating HGF secretion in CAFs significantly reduced the HGF level in the microenvironment and enhanced the efficacy of KRas inhibitors [84]. These findings highlight that a synergistic therapeutic strategy co-targeting stromal cells and tumor cells holds translational potential for the management of NSCLC.

## 3. Pathogenesis of Aberrant MET Alterations in Lung Cancer

In lung cancer, especially NSCLC, dysregulation of MET signaling has been widely implicated as an oncogenic driver [85]. Hyperactivated MET signaling to multiple downstream pathways is thought to be responsible for a range of tumorigenic cell behaviors. Anti-apoptotic and pro-survival signaling through the PI3K/AKT pathway allows for normal cell death evasion, while signaling through the ERK/MAPK pathway enhances cell motility, proliferation, and growth, contributing to metastasis and sustained tumor growth [86]. Increased amounts of HGF in the tumor microenvironment can contribute to high levels of ligand-dependent MET signaling [87]. However, HGF is not necessarily required for aberrant MET activation, as a state of MET overexpression is sufficient to enable ligand-independent activation by oligomerization of receptors [88]. Aberrant activation of MET can also occur through mutations in the *MET* gene, *MET* gene amplification, or transcriptional upregulation of *MET* without amplification. In NSCLC, all three of these mechanisms have been observed and recognized as crucial drivers [7,89].

Many reported oncogenic *MET* mutations share a common feature is the loss of exon 14 in the mRNA transcript due to aberrant pre-mRNA splicing [90]. These *MET* exon 14 skipping mutations were first reported in NSCLC in 2006, and are now known to occur in 2–4% of lung adenocarcinomas [77]. *MET* exon 14 skipping results in the deletion of the MET juxtamembrane domain containing the binding site for Cbl, an E3 ubiquitin ligase that promotes MET protein degradation. Loss of the binding site therefore results in decreased ubiquitination and degradation of the MET receptor [6,91]. In the absence of normal degradation, the resultant high-level MET expression the cell surface and sustained downstream signaling is thought to contribute to oncogenesis [7]. One analysis of tumor genomic profiles from 38,028 patients identified 221 cases with *METex14* mutations (0.6%), with a total of 126 distinct sequence variants. *METex14* mutations are detected most frequently in lung adenocarcinoma (3%) but are also frequently seen in other lung neoplasms (2.3%), brain glioma (0.4%), and tumors of unknown primary origin (0.4%) [90]. In a series of 687 Asian patients with resected NSCLC, a *METex14* alteration was shown to be a prognostic factor for poor overall survival (OS) [16]. *METex14* mutations occur in 3–4% of newly diagnosed advanced NSCLC cases [92] and is a recognized oncogenic driver [93,94]. Multiple studies have shown that patients with *METex14* mutations are characterized by strong invasiveness, poor prognosis, and a poor response to immunotherapy, with limited effectiveness of traditional treatment methods [95,96]. The emergence of highly selective MET inhibitors has broken this deadlock. Although the treatment of *METex14* mutant metastatic NSCLC varies due to differences in drug approval and clinical experience, the first-line MET TKI, such as cannot only prolong PFS but also improve the prognosis of PD-L1 < 50% and brain/bone metastases (Table 1) [97].

Gains in *MET* gene copy number (GCN) can cause cells to overexpress wild-type MET protein, which in turn allows for ligand-independent activation of the receptor. *MET* GCN gains arise from two distinct processes: polysomy and amplification [98]. High polysomy occurs when there are multiple copies of chromosome 7 in tumor cells and is secondary to factors such as chromosomal duplication [99]. In contrast, a true amplification occurs in the setting of regional gene duplication through processes indicated as breakage-fusion-bridge mechanisms [100]. In contrast to polysomy, amplification is thought to represent a state of true biological selection for *MET* activation as an oncogenic driver [77]. Notably, *EGFR* (epidermal growth factor), another proto-oncogene commonly dysregulated in NSCLC, is co-located with *MET* on chromosome 7. In all types of *MET* GCN change, copy number represents a continuous variable. An analysis of OS based on *MET* FISH status-derived GCN revealed that increased GCN is an independent adverse prognostic factor in surgically resected NSCLC, with an OS of 25.8 months for patients with *MET* ≥ 5 copies/cell, compared with 47.5 months for patients with *MET* < 5 copies/cell (*p* = 0.0045). These data support further investigation of MET-targeted therapeutic strategies in appropriately selected patients [101]. Determination of an optimal cutoff point for *MET* GCN positivity may dramatically alter its reported frequency and ultimately affect its potential to act as a predictive biomarker for benefit from MET inhibition. Primary *MET* amplification affects 2–5% of NSCLCs. However, *MET* amplification is relatively common in NSCLCs that have acquired resistance to EGFR TKIs) and appears in as many as 20% of such cases [8].

MET protein overexpression commonly occurs without gene amplification and can arise from modulation of diverse regulatory mechanisms. Transcriptional upregulation of the *MET* gene can derive from epigenetic changes in the MET locus or its regulatory genes, positive regulation by some paired box (PAX) family transcription factors (*PAX3*, *PAX5*, and *PAX8*), and inactivation of negative regulators such as *PTEN* or *P53* [102,103]. Disruption of post-transcriptional regulators, ranging from inhibitory miRNAs to proteins involved in *MET* internalization and degradation, may also promote overexpression of the MET protein [102]. MET protein overexpression, which occurs in about 30% of NSCLCs, was previously proposed as a predictive biomarker for identifying patients likely to respond to MET-targeted therapies [91]. However, its prognostic utility has come into question after multiple clinical trials have shown that MET-targeted therapies do not produce significant clinical benefits in NSCLC patients selected based on MET overexpression. In the ATTENTION (n = 307) and MET Lung (n = 499) phase III clinical trials, the MET inhibitor tivantinib, and anti-MET antibody onartuzumab, respectively, failed to achieve their primary endpoints of overall survival, despite the promising efficacy of these drugs in earlier phases of development. Both trials were selected based on positive MET IHC staining [104,105]. These failures suggest that MET IHC expression is not a good predictive biomarker for selecting patients for MET-targeted drug therapy.

**Figure 4:**
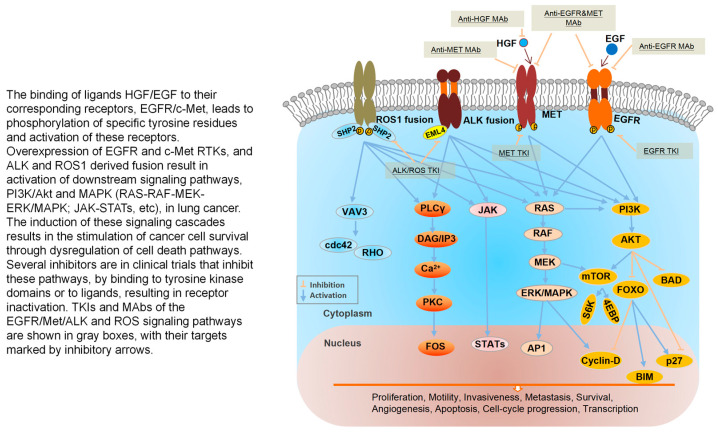
MET and RTKs share the common downstream signaling pathway in NSCLC [62,136,137,138].

In some NSCLCs, oncogenesis may be driven by synergistic cross-talk between the MET pathway and other pro-oncogenic RTK pathways, namely EGFR and anaplastic lymphoma kinase (ALK) [106]. The concurrent signaling of MET with EGFR or ALK fusion protein potentiates activation of shared downstream components, most notably the oncogenic PI3K/Akt and ERK/MAPK pathways (Figure 4). Studies of EGFR-MET interactions in NSCLC have also suggested that EGFR signaling stabilizes MET and promotes ligand-independent MET activation [107].

## 4. MET Biomarker Detection

Table 2 summarizes the current methods for detecting *MET* alterations, which include fluorescence in situ hybridization (FISH), immunohistochemistry (IHC), real-time polymerase chain reaction (RT-PCR) and next-generation sequencing (NGS) [92]. Tumors are defined as *MET* FISH-positive if at least 15% of cells show a split signal; a second confirmatory test is required if the positive signal is between 10% and 15% [108,109]. To distinguish between GCN gains caused by chromosomal polysomy and those caused by gene amplification, *MET* amplification has also been classified by using the *MET/CEP7* ratio as low (≥1.8 to ≤2.2), intermediate (>2.2 to <5), and high (≥5). Variation in classification thresholds between studies complicates comparisons of reported *MET* amplification or GCN gain relative to the underlying frequency, associated factors, and outcomes from therapy, although more rigorous data are now emerging [110].

The most widely used tool to assess the prevalence of MET protein expression in lung cancer patients has been IHC performed on formalin-fixed paraffin-embedded (FFPE) samples. MET IHC positivity requires the presence of strong granular cytoplasmic staining (3+) [120]. IHC staining for only phosphorylated MET receptors has also been proposed as a more direct evaluation of levels of receptor activation, and may be a better indicator of overall MET signaling activity [121]. It is currently widely believed that the H-score combining staining intensity and proportion is superior to the binary (positive/negative) score. Multiple studies have shown that an h score of ≥150 indicates a better response to MET inhibitors in NSCLC. However, further study will be needed to validate its diagnostic utility, especially due to the transient nature of protein phosphorylation, which may be lost during the tissue-fixing process [122]. To date, IHC reagents, procedures, and scoring methods for HGF and MET assessment have not been extensively validated, monoclonal antibodies with varying sensitivities and specificities have been used, and several different scoring systems have been investigated with a generally retrospective approach. This is reflected in the high degree of variability in the prevalence of overexpression in unselected NSCLC series, ranging from 20% to 70% for both markers [123].

NGS has been proven superior to both FISH and IHC for the sensitive detection of *MET* alterations, especially for rare mutant variants such as *MET* fusion genes. NGS detection is of great significance for targeted therapy of NSCLC. Jeffrey A Scott et al. found that patients with NSCLC who received targeted therapy after NGS had a superior survival rate, and that patients who switched therapy after NGS exhibited a superior survival rate compared to those who did not [124]. For NSCLC patients who acquire resistance to ALK or EGFR inhibitors, NGS assessment of the molecular mechanism of resistance should inform the individualized treatment strategy and selection of an appropriate subsequent treatment. FISH is the gold standard for *MET* amplification detection and is highly consistent with the accuracy of NGS analysis [125]. When NGS detection fails, FISH can be used as a supplementary verification method.

In cases where tumor tissue is insufficient for molecular testing, plasma circulating tumor DNA (ctDNA) from liquid biopsy is increasingly used as an alternative at initial diagnosis and treatment resistance [126]. Among liquid biopsy-based detection techniques, digital PCR (dPCR) is particularly suited for the precise quantitative monitoring of low-abundance *MET* molecular alterations. Su et al. demonstrated that plasma droplet digital PCR (ddPCR) exhibited substantial concordance with FISH [127].

Clinical evidence has shown that *MET* abnormalities often coexist with alterations in pathways such as *EGFR*, *KRAS*, *ALK*, and *TP53*; patients harboring such co-mutations typically show a lower response rate to single-agent targeted therapy. For instance, Xue Yang et al. utilized NGS to identify *EGFR-NTRK* dual mutations and *MET* amplification in a 58-year-old male patient diagnosed with bone-metastatic NSCLC. Subsequent treatment with a triple-inhibition regimen targeting *EGFR*, *NTRK*, and *MET* resulted in partial response (PR) and favorable tolerability [128]. This case highlights the critical role of NGS-guided precision medicine in managing patients with multiple concurrent mutations.

Currently, clinical trials employ different methods to detect MET alterations. There exists a need to establish a cross-verification process for results from different testing platforms to address the issue of false positives/false negatives in single-platform testing and standardize and optimize the detection and interpretation of the results for these MET biomarker assays to guide the clinical decisions.

## 5. Strategies to Target Aberrant MET by Mono- and Combinational Therapy

**Patient selection.** Despite the evident role of MET as an oncogenic driver, targeted therapies inhibiting the MET pathway have been met with limited success. Notably, multiple MET-inhibitory agents have recently failed phase III trials either for increasing chances of death (rilotumumab) or failing to meet their primary endpoints of OS (tivantinib and onartuzumab) [129,130]. Given the clinical benefits of tivantinib and onartuzumab for some patients in prior studies, the failure of these agents in phase III may be attributable to the inadequacy of the patient selection criteria [131]. In both trials, patient selection was based on the presence of MET overexpression indicated by positive MET IHC. MET overexpression increasingly appears to hold little predictive value for positive response to MET-targeted therapy, and a number of studies now suggest that MET overexpression does not necessarily imply a commensurate increase in receptor activation [110]. Indeed, in many MET-overexpressing and *MET*-amplified cancers, oncogenic drivers other than MET are present and may act as the primary driver [109]. Accordingly, a major focus of research efforts is the identification of better diagnostic biomarkers for cancers that are in fact MET-driven, and therefore likely to respond to MET inhibitors. In NSCLC, high *MET* amplification (*MET/CEP7* ≥ 5), but not low or intermediate amplification, is associated with response to the MET TKI crizotinib [132]. *MET* exon 14 skipping mutations in NSCLC also predict modest to strong responses to the MET TKIs crizotinib, tepotinib, and capmatinib [90,133]. Currently, only crizotinib is recommended by the NCCN for high-level *MET* amplification or *MET* exon 14 skipping mutations [134].

**MET inhibitors.** As illustrated in Figure 4, three strategies have been devised to inhibit the HGF/-MET signaling pathway: anti-HGF monoclonal antibodies, anti-MET monoclonal antibodies, and small molecule MET TKIs. Monoclonal antibody therapy is divided into anti-MET antibodies (e.g., onartuzumab, emibetuzumab, and SAIT301) and anti-HGF antibodies (e.g., ficlatuzumab and rilotumumab) [82,135]. Anti-MET and anti-HGF antibodies bind to their respective targets to occlude the binding of MET to HGF, and consequently, the majority of these antibodies inhibit only ligand-dependent MET signaling. However, some anti-MET antibodies, such as emibetuzumab and SAIT301, have also been shown to promote internalization and degradation of MET, and thus inhibit both ligand-independent and ligand-dependent HGF/MET signaling [78]. In phase II trials, onartuzumab plus erlotinib was associated with improved PFS and OS in an MET-positive population of NSCLC patients [131].

The binding of ligands HGF/EGF to their corresponding receptors, EGFR/c-Met, leads to phosphorylation of specific tyrosine residues and activation of these receptors. Overexpression of EGFR and c-Met RTKs, and ALK and ROS1 derived fusion result in activation of downstream signaling pathways, PI3K/Akt and MAPK (RAS-RAF-MEK-ERK/MAPK; JAK-STATs, etc.), in lung cancer. The induction of these signaling cascades results in the stimulation of cancer cell survival through dysregulation of cell death pathways. Several inhibitors are in clinical trials that inhibit these pathways, by binding to tyrosine kinase domains or to ligands, resulting in receptor inactivation. TKIs and MAbs of the EGFR/Met/ALK and ROS signaling pathways are shown in gray boxes, with their targets marked by inhibitory arrows.

Small molecule MET TKIs can be subdivided into multikinase and selective MET inhibitors. Examples of multikinase MET inhibitors are crizotinib, which also inhibits ALK and ROS1, and cabozantinib, which also inhibits VEGFR2 and RET. Selective MET inhibitors include capmatinib, savolitinib, and tivantinib. In some cases, multikinase inhibitory activity may be desirable if more than one of the kinase targets shows signs of oncogenic activation, although a similar effect may be achieved by a combination of specific inhibitors [139]. Theoretically, multikinase inhibitors could also reduce the incidence of certain types of resistance by inhibiting potential bypass pathways. More selective inhibitors, however, typically produce less off-target toxicity [139]. The advantages and drawbacks of each category are not yet well understood.

While all small molecule MET TKIs inhibit the enzymatic activity of the MET tyrosine kinase, they differ in how they bind to the tyrosine kinase domain. ATP-competitive MET inhibitors, which can be classified by binding mode as type I or type II, bind at the kinase hinge region and occupy the ATP binding site. Type I inhibitors bind to the active form of the kinase and may be relatively selective (type Ib) or non-selective (type Ia), while type II inhibitors bind to the inactive form of the kinase and often possess some multikinase activity. The distinct binding regions targeted by each binding mode may partially account for the differential effectiveness of type I and II MET inhibitors against specific resistance mutations affecting residues near the active site [140]. The ATP non-competitive MET inhibitor tivantinib is fairly distinct from the other agents and is notable for its highly selective allosteric inhibition of the MET kinase [140] (Table 3).

In current clinical practice, MET inhibitors are predominantly used as salvage therapy after the progression of TKIs treatment. However, an increasing evidence suggests that early combination with MET inhibitors is more likely to prevent drug resistance and prolong the benefits for patients. Early combination therapy can block the potential activation of the MET pathway prior to the emergence of resistant clones, thereby averting the onset of treatment resistance. Additionally, in the early disease stage, tumor burden is relatively low, and the TIME has not yet fully deteriorated. consequently, combination therapy is more prone to exerting synergistic effects and significantly prolonging the duration of treatment response. Notably, early combination therapy is not applicable to all patients with TKIs-driven tumors. It is necessary to precisely screen the beneficiary population to balance efficacy and safety. The multi-platform combined detection using NGS, IHC and liquid biopsy facilitates the identification of biomarkers predictive (such as MET amplification of GCN, the threshold of H-score, the cutoff value of HGF concentration, etc.) of response to early combination therapy.

***MET* amplification.** Several MET inhibitors have been investigated for NSCLC patients with *MET* amplification. Crizotinib is a type I ATP-competitive TKI originally developed to target *MET* and has since been found to also inhibit *ALK* and *ROS1*. In at least one case, a NSCLC patient with *MET* amplification but no *ALK* fusion gene achieved rapid and sustained remission with the administration of crizotinib, indicating that Crizotinib may be clinically useful as an MET inhibitor [141]. A 2014 study reported on the efficacy and safety of crizotinib in advanced *MET*-amplified NSCLC. FISH was used to determine the *MET* amplification status of enrolled patients, who were then divided into three groups according to the *MET/CEP7* ratio: low (≥1.8 to ≤2.2), medium (>2.2 to <5), and high (≥5). 13 patients met MET/*CEP7* criteria to receive crizotinib, 12 patients were evaluable for response. This study is part of an ongoing phase I study of crizotinib (NCT00585195) [142].

Capmatinib is a type I ATP-competitive TKI and selective to *MET*. In clinical trial NCT01610336, a combination of capmatinib with gefitinib, an EGFR TKI, showed encouraging clinical activity in EGFR TKI-resistant NSCLC patients particularly in patients with high *MET* GCN. Partial responses were seen in 12/65 evaluable patients (ORR 18%), and 40/65 (62%) patients had stable disease (SD); 10/53 patients with IHC 3+ or IHC 2+ and GCN ≥ 5 had PRs (ORR 19%), and 7/23 patients with GCN ≥ 6 had PRs (ORR 30%) [143].

***MET* exon 14 skipping mutations.** In the ongoing PROFILE 1001 subgroup study evaluating crizotinib monotherapy in 69 advanced NSCLC patients with *MET* exon 14 alterations, modest clinical activity was observed: the ORR was 32% (95% CI: 21, 45) in 65 response-evaluable patients, the median duration of response was 9.1 months, and median time to response was 7.6 weeks (range: 3.7–16.3).28 PFS and OS data were not mature by the data cutoff date in June 2018, at which time 35% of patients had died, and 40.6% were still in follow-up. Median PFS is estimated at 7.3 (95% CI: 5.4, 9.1) months, and median OS is estimated at 20.5 (14.3–21.8) months. No new safety concerns were observed. Based on these data, the US FDA granted breakthrough designation to crizotinib as a second-line treatment for NSCLC patients with *MET* exon 14 skipping mutations earlier this year. Crizotinib has demonstrated significant efficacy in improving survival outcomes among patients with *MET* exon 14 skipping mutations, serving as a pivotal therapeutic option. Furthermore, its clinical success has accelerated the development and translation of next-generation MET inhibitors with enhanced potency and selectivity.

Several other MET inhibitors in clinical trials have shown activity against *METex14* mutations. In the opening phase II trial (VISION) in advanced NSCLC patients with *METexon14* skipping alterations, tepotinib had an ORR of 51.4% (95% CI, 45.8, 57.1) and median DOR of 18.0 months (95% CI, 12.4, 46.4) in 313 patients by an independent review committee at data cutoff in November 2022 [96]. Perhaps the most encouraging development was presented in 2024: in the final results of 160 patients (60 were newly diagnosed with the disease, and 100 had already received one or two therapies) with advanced NSCLC harboring *MET* exon 14–skipping mutations in the phase II GEOMETRY mono-1 trial, capmatinib (INC280) had the ORR of 68% in treatment-naive patients, which was one of the highest overall response rates reported thus far with MET tyrosine kinase inhibitors. And it had the ORR of 41% in the second-line or third-line setting and 52% in the second-line setting. These data further support that patients with *MET* exon 14–skipping mutations might respond better to capmatinib [144].

***MET* fusions gene.** *MET* fusion is a rare genomic event, advances in detection technologies helps enable identification of various *MET* fusion partner genes. At present, The standard treatment regimen for *MET* fusion in NSCLC remains unclear, but distinguishing *MET* fusion partners is crucial for subsequent treatment. Sun et al. and Ganlu Ouyang et al. reported crizotinib/savolitinib treatment could cause partial response *EML4-MET* fusion [38,39]. A case report of patients with *CD47-MET* fusion NSCLC also demonstrated that crizotinib was an effective treatment for patients with this condition [40]. A report on a case of NSCLC with *EGFR G719D/L861Q* mutation and *CUX1-MET* fusion revealed that *MET* fusion may constitute a key mechanism underlying acquired resistance to targeted therapy in patients with rare *EGFR* mutations [145]. Zhuo et al. found that the most frequent breakpoint within the *MET* fusion genes are located in intron 14, while exons 15–21 are preserved [146]. The mechanism by which these exons cross the entire kinase region to cause *EML4-MET* fusion mutations may be similar to the *MET* exon 14 skipping, so the treatment of *MET* exon 14 skipping may also be applicable to *MET* fusion gene. However, the response of different *MET* fusion partners to treatment is heterogeneous; therefore, future studies should investigate the specific mechanism of action of this fusion gene.

Multiple studies have indicated that for newly diagnosed NSCLC patients with *MET* fusion, highly selective type I MET inhibitors (e.g., camatinib, savolitinib) or multi-targeted tyrosine kinase inhibitors (e.g., crizotinib) are the preferred first-line therapeutic options. Moreover, treatment selection can be further optimized based on the specific fusion partner subtype; for instance, crizotinib or capmatinib is preferred for patients with *KIF5B-MET* fusion. In the event of disease progression following monotherapy with an MET inhibitor, it is imperative to clarify the underlying resistance mechanism via NGS and consider switching to an alternative MET inhibitor of a different type. If resistance is driven by bypass pathway activation (e.g., *EGFR* amplification), a combination regimen incorporating the original MET inhibitor plus a targeted agent against the bypass pathway is recommended. Furthermore, for the special populations with brain metastases, priority should be given to MET inhibitors with proven blood–brain barriers (e.g., tepotinib, crizotinib). Alternatively, a combined strategy of MET inhibitor therapy plus whole-brain radiotherapy may be adopted.

**Figure 5:**
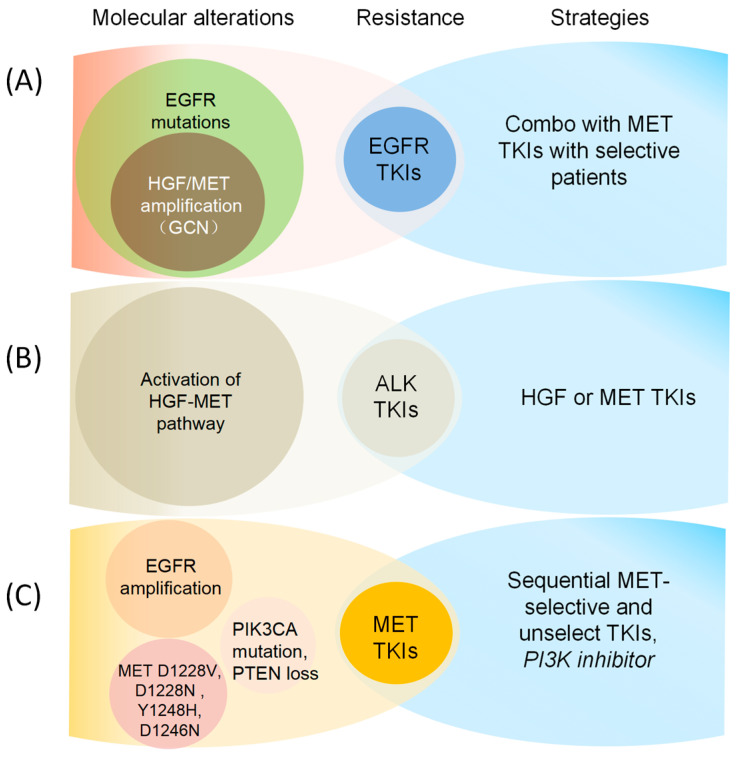
Molecular Mechanisms and strategies for overcoming resistance to EGFR and MET TKIs, including (**A**) EGFR mutation combined with HGF/MET amplification (GCN) confers resistance to EGFR TKIs [8,15,112,148]; (**B**) Activation of the HGF-MET pathway leads to resistance of ALK TKIs [149,150,151]; (**C**) EGFR amplification/MET mutation/PIK3CA mutation/PTEN loss confers resistance to MET TKIs [30,152,153,154].

**Combinational studies.** Combinations of MET inhibitors and other agents have been investigated as treatments for NSCLC. Among these, the most well-studied combination has been MET inhibitors with EGFR inhibitors (Table 4). In EGFR-activated NSCLC, activation of the MET pathway is a mechanism of both primary (de novo) and acquired resistance to EGFR inhibitors, particularly EGFR tyrosine kinase inhibitors (TKIs). A substantial overlap is present between MET and EGFR downstream signaling targets and includes important pro-oncogenic pathways such as PI3K/AKT and ERK/MAPK (Figure 5) [81]. MET activation may therefore bypass EGFR inhibition by compensatory signaling to these shared downstream pathways. Coincident MET and EGFR activation may synergistically enhance the signal strength of their common pro-survival, pro-proliferation pathways, thus confer a general selective advantage to tumor cells [147,148]. MET and EGFR inhibitor combination strategies seek to exploit cellular dependence on this oncogenic synergy, as simultaneous inhibition of both targets may also produce synergistic anti-tumor effects.

**Table 4:** Clinical trials evaluating the combination of targeting MET and EGFR.

Clinical Trials	MET Ihibitors	EGFR Inhibitors	Target Cohorts	No. Patients	Phase	Comments (Reference)
NCT00854308	Onartuzumab	Erlotinib	EGFR-mutant advanced NSCLC	137	II	Onartuzumab plus erlotinib was associated with improved PFS and OS in the MET-positive population [116].
NCT00777309	Tivantinib	Erlotinib	Previously treated patients with EGFR TKI–naive advanced NSCLC	167	II	No significant improvements in PFS; Cohort with KRAS mutations achieved a PFS HR of 0.18 (*p* = 0.002) [46].
NCT01244191 (MARQUEE)	Tivantinib	Erlotinib	Locally advanced or metastatic non-squamous NSCLC who had received one or two lines of prior systemic therapy	1048	III	Modest improvement in PFS, no improvement in OS [155].
NCT01377376 (ATTENTION)	Tivantinib	Erlotinib	Asian nonsquamous NSCLC patients with wild type EGFR	460	III	Tivantinib plus erlotinib might improve PFS than erlotinib alone but did not demonstrate an improvement in OS in nonsquamous NSCLC patients with WT-EGFR [104].
NCT01708954	Cabozantinib (XL184)	Erlotinib	EGFR-wild type NSCLC	125	II	Cabozantinib and cabozantinib plus erlotinib significantly improved PFS over erlotinib alone [156]; There were no responses in the combination arm of phase II in patients with acquired resistance to erlotinib [157].
NCT01866410	Cabozantinib	Erlotinib	EGFR-Mutant NSCLC who had progressed to EGFR TKIs	37	I	Median PFS was 3.7 months. Combination of erlotinib and cabozantinib demonstrates activity in a highly pretreated population of patients with EGFR mutation and progression on EGFR TKI [158].
NCT01121575	Crizotinib	Dacomitinib	Progression after at least one line of chemotherapy or targeted therapy	70	I	Limited antitumor activity, substantial toxicity, diarrhea, rash, and fatigue, but has only modest clinical efficacy [159].
NCT01911507	Capmatinib (INC280)	Erlotinib	MET expressing NSCLC	44	I	Showed encouraging clinical activity in EGFR TKI-resistant NSCLC pts [160].
NCT01610336	Capmatinib (INC280)	Gefitinib	EGFR-mutant, cMET-positive (cMET+)	161	Ib/II	Showed encouraging clinical activity in EGFR TKI-resistant NSCLC pts, particularly in pts with high cMET GCN [143].
NCT02335944	Capmatinib	EGF816	MET amplification, acquired resistance to osimertinib and rocelitinib	180	II	Preclinical study showed advantage of inhibiting T790M, targets acquired resistance and reduce toxicities [161].
NCT01982955	Tepotinib	Gefitinib	Asian patients with c-Met-positive/EGFR-mutant NSCLC	70	1b/II	Improved anti activity for tepotinib plus gefitinib compared with standard chemotherapy in patients with EGFR-mutant NSCLC and MET amplification [162].
NCT02143466	Savolitinib (AZD609)	Osimertinib	EGFR-mutant advanced NSCLC who have progressed following therapy with an EGFRTKI; Metastatic NSCLC with MET-mediated resistance to EGFR TKI	308	Ib	Savolitinib (800 mg once daily) and osimertinib (80 mg once daily); Objective response rate was 44% (22% to 69%) [56], response 36 W then progress; 1 Showed advantage of inhibiting T790M, targeted acquired resistance and reduce toxicities with acceptable risk-benefit [163].
NCT02609776 (CHRYSALIS)	Amivantamab	Lazertinib	Treatment-naïve and osimertinib (osi)-relapsed patients (pts) with EGFRm NSCLC	780	I	The combination of amivantamab and lazertinib yielded responses in 36% of chemotherapy-naïve pts who progressed on osi [61,164].
NCT00965731	Crizotinib	Erlotinib	Advanced NSCLC harbored activating EGFR mutations	27	I/II	Crizotinib (150 mg twice daily) and erlotinib (100 mg once daily), achieved confirmed partial responses (2/27); diarrhea, rash, decreased appetite, and fatigue [50].
NCT05009836 (SANOVO)	Savolitinib	Osimertinib	Patients with EGFRm+/MET+ NSCLC, and Carrying EGFR mutations sensitive to EGFR-TKI	320	III	No results posted yet.
NCT05015608 (SACHI)	Savolitinib	Osimertinib	NSCLC (stage IIIB, IIIC or IV), MET amplification after disease progression following the first-line therapy and/or EGFR-TKI.	250	III	No results posted yet.
NCT05163249	Savolitinib	Osimertinib	EGFRm+ NSCLC harboring an EGFR TKI sensitivity mutation, and with MET amplification	44	II	No results posted yet.
NCT05261399 (SAFFRON)	Savolitinib	Osimertinib	NSCLC with at least one documented sensitizing EGFR mutation and with MET overexpression	324	III	No results posted yet.
NCT06106802	Tepotinib	Lazertinib	MET Overexpressed or Amplified after Lazertinib Treatment in EGFR Mutant NSCLC	47	II	No results posted yet.
NCT04606771	Savolitinib	Osimertinib	Comparing Savolitinib plus Osimertinib vs. Savolitinib plus Placebo in EGFRm+ and MET amplified advanced NSCLC	30	II	No results posted yet.
NCT06343064	Vebreltinib (PLB1001)	PLB1004	Vebreltinib plus PLB1004 in EGFR-mutated, Advanced NSCLC With MET Amplification or MET Overexpression Following EGFR-TKI	156	II	No results posted yet.
NCT04868877	MCLA-129	MCLA-129	MCLA-129, a Human Anti-EGFR and Anti-c-MET Bispecific Antibody	380	II	No results posted yet.
NCT04992858	ningetinib	gefitinib	Ningetinib + gefitinib show activity in EGFRmt, MET-amp, AXL-overexpressed NSCLC.	80	II	Ningetinib plus gefitinib in EGFR-mutant non-small-cell lung cancer with MET and AXL dysregulations: A phase 1b clinical trial and biomarker analysis [165].
NCT06106802	Tepotinib	Lazertinib	EGFR Mutant NSCLC in MET Overexpressed or Amplified Who Progressed After Lazertinib Treatment	47	II	No results posted yet.
NCT06574347	Vebreltinib	PLB1004	EGFRm+/MET+ Locally Advanced or Metastatic NSCLC	120	II	No results posted yet.
NCT06970782	Vebreltinib	PLB1004	EGFR Mutations, MET Amplification and/or Overexpression, locally advanced or metastatic NSCLC following EGFR-TKI treatment failure	278	III	No results posted yet.
NCT06962865	RC108	Furmonertinib	EGFR-Mutated Combined MET-Positive Unresectable Locally Advanced or Recurrent Metastatic NSCLC	80	II	No results posted yet.
NCT07109531	ASKC202	Limertinib	Locally advanced or metastatic NSCLC with MET amplification/overexpression after failure of EGFR inhibitor therapy	286	III	No results posted yet.
NCT07087223	Vebreltinib	Furmonertinib	Locally Advanced or Metastatic NSCLC Patients With c-Met Amplification After EGFR-TKI Failure	42	Ib/II	No results posted yet.

In the context of acquired resistance to EGFR TKIS, MET activation most frequently occurs via amplification of the *MET* gene. *MET* amplification was first identified in 2007 as a cause of developed resistance to the EGFR TKI gefitinib [8]. Around the same time, *MET*-amplified cell lines exhibited sensitivity to MET inhibition [166]. In lung cancer, *MET* amplification is now recognized as the second most common mechanism of acquired resistance to EGFR TKIs, after the acquisition of the *EGFR T790M* mutation. Approximately 20% of NSCLCs with acquired resistance to EGFR TKIs have *MET* amplification [93,167]. MET activation is also implicated as a potential de novo mechanism of resistance: one 2010 study reported that MET protein expression and phosphorylation were associated with de novo resistance to EGFR TKI therapy in NSCLC patients harboring activating *EGFR* mutations [168].

Despite the sound rationale for combining EGFR TKIs and MET inhibitors, some trials have encountered problems. As previously noted, two major phase III clinical trials investigating erlotinib with either tivantinib or onartuzumab failed to meet their primary endpoints of OS, largely due to inadequate patient selection criteria based on MET IHC positivity. Additionally, issues associated with the pharmacological interactions and overlapping toxicity profiles of the EGFR TKI erlotinib and the MET TKI crizotinib, particularly with respect to diarrhea, suggest that these drugs may not be suitable combinatorial partners. The limited efficacy further argues against the evaluation of this combination in unselected patients [169]. Matthew G Krebs et al. reported that treatment-emergent adverse events (TEAEs) related to EGFR and MET inhibition—including dermatologic AEs, stomatitis, and peripheral edema—accounted for the majority of dose interruptions and reductions in the study cohort [170]. The most frequently observed TEAEs were rash and IRRs, with 77 participants (79%) and 70 participants (72%) reporting these events, respectively. Among them, the number of patients who interrupted, reduced or stopped medication due to treatment-related adverse reactions was 27 (28%), 11 (11%) and 9 (9%), respectively.

In contrast, some encouraging results have been reported with the combination of the highly potent, selective MET inhibitor capmatinib (INC280) with the EGFR-TKI gefitinib. A combination of a full single agent dose of gefitinib (250 mg daily) with capmatinib (400 mg twice daily) has shown acceptable toxicity and no pharmacologic interactions. The response rate to the combination was 31% (28 of 90 cases), with the best results seen in patients with an *MET* copy number gain of at least six, with a response rate of 50% (16 of 32 cases) reported. Similarly, favorable results have been reported for the combination of capmatinib with erlotinib, as well as the combination of gefitinib with tepotinib, another MET inhibitor [143,162].

The ongoing, multi-arm, phase Ib TATTON study investigates the potent and highly selective MET-TKI savolitinib (600 mg/300 mg) combined with the EGFR-TKI osimertinib (80 mg) for selected patients with MET-positive advanced NSCLC and resistance to previous EGFR-TKI treatment. As of Jan 2023, results from TATTON show an acceptable safety profile and promising anti-tumor activity for the combination. In the cohort with disease progression on a no prior third-generation EGFR-TKI, the ORR was 65% (33 of 51 patients), and the median duration of response was 10.7 months. In the cohort with disease progression on a prior third-generation EGFR-TKI, the ORR was 33% (23 of 69 patients) and the median duration of response was 9.5 months. The TATTON study also demonstrated no appreciable difference in antitumor efficacy among the evaluated dose cohorts of savolitinib, a conclusion that is substantiated by the progression-free survival (PFS) data [171]. The results cutoff date in June 2025 from SAVANNAH showed the combination of savolitinib plus osimertinib demonstrated a high, clinically meaningful and durable response in patients with EGFR-mutated advanced NSCLC with MET IHC3+/≥90% and/or FISH10+ status, and the ORR was 56.3 by investigator assessment [172].

In other promising preclinical studies, a selective MET inhibitor SGX-523 combined with either first or third generation EGFR-TKIs led to a dramatic regression of EGFR-TKI resistant tumors and delayed the emergence of drug resistance [173,174].

## 6. MET TKI Resistance

Several mechanisms of resistance to MET inhibitors have been investigated in NSCLC, including *EGFR* amplification and emergence of secondary *MET* mutations in the tyrosine kinase domain (Figure 5C). Clinically described *MET* mutations associated with acquired MET inhibitor resistance (*D1228V*, *D1228N*, *Y1248H* and *D1246N*) alter residues near the active site involved with drug binding. Interestingly, these mutations confer resistance to type I but not type II MET inhibitors, likely due to the difference in binding regions utilized [154,175].

When a target mutation occurs in MET kinase domain, it is called type I resistance [176]. Selected patients who acquire resistance to a type I MET inhibitor could benefit from switching to a type II MET inhibitor [20]. MET type II resistance, on the other hand, develops resistance to MET inhibitors through mechanisms such as bypass activation, at which point the type I/II MET inhibitors lose their inhibitory effect. In vitro studies have also placed *EGFR* amplification as a cause of de novo MET inhibitor resistance in MET-dependent NSCLC cell lines [177]. Sequential therapy with distinct MET inhibitors could potentially overcome type- or drug-specific acquired resistance. In addition, multikinase MET inhibitors with differing kinase target profiles could conceivably overcome specific resistance mechanisms involving its other kinase targets; however, the implications of multikinase activity for treatment are not well understood. Treatment of MET TKI-resistant NSCLC will require case-by-case identification of the resistance mechanism and tailored treatment strategies.

## 7. Immunotherapy

A substantial percentage of MET-activated NSCLCs express the immunosuppressive ligand PD-L1. Among *MET*-amplified NSCLCs, the reported prevalence of PD-L1 IHC expression is as high as 34.6% at a >5% cutoff and 18.6% at a ≥50% cutoff. In NSCLC with *METex14* alterations, the PD-L1 IHC expression rate is 61% at a >1% cutoff, and 44% at a ≥50% cutoff. The frequency of PD-L1 expression in these tumors has prompted exploration into treatment with immune checkpoint inhibitors (ICIs) against PD-L1 and PD-193.

However, monotherapy with PD-L1/PD-1 blockades has thus far produced disappointing results in lung cancer patients with *METex14* alterations. A retrospective study of 15 such patients, variously receiving nivolumab, pembrolizumab, atezolizumab, durvalumab, or ipilimumab with nivolumab, reported an ORR of 13%. Patients with ≥50% PD-L1 in tumors had an ORR of 33%, and those with 0% PD-L1 in tumors had an ORR of 20%. The response rate to ICIs seen in this study is relatively low compared to that of targeted MET inhibitors (crizotinib, tepotinib, and capmatinib) for *METex14*-altered NSCLC. This modest response rate may owe in part to the low median tumor mutational burden (TMB) of *METex14*-altered lung cancers relative to unselected NSCLCs; tumors with a low TMB often display fewer neoantigens recognizable by lymphocytes and are less likely to respond to immunotherapy.

MET activation may also in itself contribute to ICI resistance. While the immunomodulatory functions of the MET pathway are not well characterized, it appears to play an overall immunosuppressive role in cancers, primarily by inducing an increase in Treg cells and increases in IL-10 and TGF-β. Furthermore, MET signaling has been shown to directly upregulate PD-L1 expression in several lung adenocarcinoma cell lines. In NSCLCs, PD-L1 expression is also significantly and positively correlated with MET expression.

*METex14* alterations and PD-L1 overexpression engage in a bidirectional rather than unidirectional regulation, further intensifying immune escape. Specifically, *METex14* aberrations remodel the tumor immune microenvironment through multiple mechanisms and trigger distinct pro-immunosuppressive signaling cascades. Beyond its canonical role as an immunosuppressive checkpoint molecule, PD-L1 also participates in the core signaling pathways governing tumor cell proliferation. Consequently, monotherapy targeting the PD-1/PD-L1 axis fails to abrogate *METex14*-driven tumor progression or disrupt the synergistic immune evasion machinery orchestrated by these two factors—a major contributor to the limited efficacy of ICIs in this patient population. Therefore, combination therapy of ICIs with targeted MET inhibitors remains an attractive avenue of investigation and may be able to overcome ICI resistance conferred by overactive, immunosuppressive MET signaling [178]. However, caution should be exercised with regard to toxicity, as simultaneous inhibition of multiple immunoregulatory targets could also increase the likelihood of serious immune-related adverse events. There also exists a need to identify and validate good predictive biomarkers for the positive response to ICI therapy. PD-L1, in particular, is increasingly recognized as a highly imperfect predictor of ICI treatment outcome. Interpretation of PD-L1 status could be further confounded in MET-activated tumors since it may be unclear when PD-L1 expression is a secondary consequence of MET signaling or offers a crucial selective advantage in escaping immune surveillance.

## 8. Conclusions

Activation of the MET pathway represents both an oncogenic driver and a promising therapeutic target in NSCLC. Historically, drug development has focused primarily on MET TKIs and, to a lesser extent, monoclonal antibodies. Although many MET inhibitors have not met their primary endpoints in phase III trials, they continue to show clinical benefit in specific patient subgroups with MET-driven alterations, particularly *MET* amplification and *MET* exon 14 skipping mutations. Future progress will depend on improving patient selection through the development of reliable companion diagnostics and a deeper understanding of the mechanistic differences among MET inhibitors. Combination therapy strategies have shown greater clinical promise than monotherapy by mitigating resistance and enhancing efficacy. Ongoing research in these areas is essential to fully integrate MET-targeted therapies into routine clinical practice and expand treatment options for patients with NSCLC.

## Figures and Tables

**Table 1:** Clinical trials evaluating the *MET* Exon14 (Targeting Exon 14 Skipping).

Agent		Specimen	MET Testing	N	Brain (N) Metastasis	ORR% (95%CI)	DOR (Months)	PFS (Months)
Clinical Trail	Capmatinib (GEOMETRY study)	Tissue	RT-PCR	97		1L-67.9 (47.6, 84.1)	1L-11.1 (5.55, NE)	1L-9.7 (5.5,13.86)
				1L-28	1L-3			
				2/3L-69	2/3L-11	2/3L-40.6 (28.9, 53.1)	2/3L-9.7 (5.55, 12.98)	2/3L-5.4 (4.2, 6.97)
	Tepotinib (VISION study)	Liquid	NGS	87 1L-33 2/3L-54	8	Liquid-50 (35.2, 64.8)	Liquid-12.4 (5.8, NE)	Liquid-9.5 (6.7, NE)
						1L-58.8 (32.9, 81.6)		
						2L-53.3 (26.6, 78.7)		
						≥2L-45.2		
		Tissue	NGS			Tissue-45.1 (31.1, 59.7)	Tissue-15.7 (9.0, NE)	Tissue-10.8 (6.9, NE)
						1L-44.4 (21.5, 69.2)		
						2L-50 (26, 74)		
						≥2L-45.5		
	Crizotinib	Tissue-local		65	NA	32 (21, 45)	9.1 (6.4, 12.7)	7.3 (5.4, 9.1)
		Prospective central tissue						
		Liquid ctDNA						
	Savolitinib	Tissue		29	5	54.8	NA	NA
First-line Therapy	Pembro (PD-L1 >= 50%)	Tissue	IHC	154	18	44.8 (36.8, 53)	NR (1.9+, 14.5+)	10.3 (6.7, NE)
	Carbo/pem/pembro (non-squam)					47.6	11.2	8.8
	Capmatinib	Tissue				67.9	11.1	9.7
	Tepotinib	Tissue				44.4	15.7	10.8
		Blood				58.8	12.4	9.5

NA: Not Applicable; NE: Not Evaluated.

**Table 2:** Methods for detecting MET Alterations in lung cancer.

Genetic Alteration	Common Genotypes	Prevalence	FISH	IHC	NGS	PCR
MET amplification	Somatic copy number	5.2% (12 of 230) [89]			Yes	
	MET copy number ≥ 5 (polysomy + gene amplification)	11.1% (48 of 435) [111]	Yes		Yes	
	MET copy number ≥ 5 (polysomy only)	4.1% (18 of 435) [111]	Yes			
	MET copy-number > 3	5.6% (12 of 213) [9]				Yes
	MET/CEP7 ratio ≥ 5	1.0% (4 of 392) [16,101]				
	MET amplification	1.4% (2 of 148) [10]	Yes		Yes	
	MET amplification	3.27% (82 of 2507) [112]			Yes	
Exon 14 skipping alterations	MET splice mutations (JM domain deleted)	3.3% (7 of 211) [10]			Yes	
	Exon 14 skipping alterations	4.3% (10 of 230) [89]			Yes	
	Exon 14 skipping alterations	3% (131 of 4402) [90]			Yes	
	Exon 14 skipping alterations	1.7% (3 of 178) [10]			Yes	
	Exon 14 skipping alterations	2.6% (10 of 392) [16]			Yes	
	Exon 14 skipping alterations	1.1% (125 of 11,306) [113] 1.1%, (175 of 18,112) [31]			Yes	
Protein overexpression	MET/p-MET expression in NSCLC	37% ≥ 2+ [110]; 61% ≥ 2+ [114]		Yes		
	MET/p-MET expression in ADC	35% [115]; 67% ≥ 2+ [114]; 72% [4]		Yes		
	MET/p-MET expression in SCC	38% [4]		Yes		
METi-resistant mutations	D1228N in the MET kinase domain [116]	NA			Yes	
	Y1230H in the MET activation loop [117]	NA			Yes	
	L1195V, D1228N/H/Y/E, Y1230C/H/N/S, and a double-mutant within codons D1228 and M1229 in MET TKD [19]	NA			Yes	
MET fusions	KIF5B–MET [118]	NA			Yes	
	HLA-DRB1-MET [119]	NA			Yes	
	EML4-MET [39]	NA			Yes	
	CD47-MET [40]	NA			Yes	
	TFG-MET [41]	NA			Yes	
	HLA-DQB2-MET [42]	NA			Yes	

NA: Not Available.

**Table 3:** Approval Status and Key Characteristics of Type I/II MET Inhibitors.

Inhibitor Type	Drug	Approval	Remark
Type I MET inhibitors	Capmatinib	FDA: 2020.05 NMPA: 2024.09	High selectivity, effective against METex14 skipping mutations and MET amplification
	Tepotinib	FDA: 2021.02 NMPA: Not approved	It can penetrate the blood–brain barrier and has good tolerance
	Savolitinib	FDA: Not approved NMPA: 2021.06	The first drug approved for Chinese patients with METex14 skipping mutations
	Crizotinib	FDA: 2011.08 NMPA: 2013.01	Multi-target inhibitors, low specificity
Type II MET inhibitors	Cabozantinib	FDA: 2012.11 (Medullary thyroid carcinoma), 2016.04 (Advanced renal cell carcinoma) NMPA: 2021.03 (Liver cancer), Not approved (NSCLC)	Multi-target coverage such as MET, VEGFR2, and AXL

## Data Availability

No new data were created or analyzed in this study. Data sharing is not applicable to this article.

## Abbreviations

The following abbreviations are used in this manuscript:

ALK	anaplastic lymphoma kinase
CAFs	cancer-associated fibroblasts
CBL	Casitas B-lineage lymphoma protein
ctDNA	circulating tumor DNA
dPCR	digital PCR
ddPCR	droplet digital PCR
EGFR	epidermal growth factor
ERK/MAPK	Erk/mitogen-activated protein kinase
FFPE	formalin-fixed paraffin-embedded
FISH	in situ hybridization
GCN	gene copy number
Grb2	growth factor receptor-bound protein 2
GAB1	Grb2-associated binder
HGF	hepatocyte growth factor
HGFR	hepatocyte growth factor receptor
ICIs	immune checkpoint inhibitors
IHC	immunohistochemistry
IPT	Immunoglobulin-plexin-transcription
JM	juxtamembrane
MET	Mesenchymal-Epidermal Transition
NSCLC	Non-small cell lung cancer
NGS	Next-generation sequencing
OS	overall survival
PAX	paired box
PI3K/AKT	phosphoinositide 3-kinase/Akt pathway
PSI	plexins-semaphorin-integrin
PR	partial response
RT-PCR	real-time polymerase chain reaction
Scr2	Steroid receptor coactivator 2
SEAM	sema homology region
SF	scatter factor
SHC	Src homology 2 domain containing
STAT3	signal transducer and activator of transcription 3
TEAEs	treatment-emergent adverse events
TK	tyrosine kinase
TKIs	tyrosine kinase inhibitors
TMB	tumor mutational burden

## References

[1] Cooper C.S., Park M., Blair D.G., Tainsky M.A., Huebner K., Croce C.M., Vande Woude G.F. (1984). Molecular cloning of a new transforming gene from a chemically transformed human cell line. Nature.

[2] Weidner K.M., Behrens J., Vandekerckhove J., Birchmeier W. (1990). Scatter factor: Molecular characteristics and effect on the invasiveness of epithelial cells. J. Cell Biol..

[3] Rong S., Bodescot M., Blair D., Dunn J., Nakamura T., Mizuno K., Park M., Chan A., Aaronson S., Vande Woude G.F. (2023). Tumorigenicity of the met Proto-Oncogene and the Gene for Hepatocyte Growth Factor. Mol. Cell. Biol..

[4] Ichimura E., Maeshima A., Nakajima T., Nakamura T. (2005). Expression of c-met/HGF Receptor in Human Non-small Cell Lung Carcinomas in vitro and in vivo and Its Prognostic Significance. Jpn. J. Cancer Res..

[5] Siegfried J.M., Weissfeld L.A., Luketich J.D., Weyant R.J., Gubish C.T., Landreneau R.J. (1998). The clinical significance of hepatocyte growth factor for non–small cell lung cancer. Ann. Thorac. Surg..

[6] Ma P.C., Kijima T., Maulik G., Fox E.A., Sattler M., Griffin J.D., Johnson B.E., Salgia R. (2003). c-MET Mutational Analysis in Small Cell Lung Cancer: Novel Juxtamembrane Domain Mutations Regulating Cytoskeletal Functions. Cancer Res..

[7] Kong-Beltran M., Seshagiri S., Zha J., Zhu W., Bhawe K., Mendoza N., Holcomb T., Pujara K., Stinson J., Fu L. (2006). Somatic Mutations Lead to an Oncogenic Deletion of Met in Lung Cancer. Cancer Res..

[8] Engelman J.A., Zejnullahu K., Mitsudomi T., Song Y., Hyland C., Park J.O., Lindeman N., Gale C.-M., Zhao X., Christensen J. (2007). MET Amplification Leads to Gefitinib Resistance in Lung Cancer by Activating ERBB3 Signaling. Science.

[9] Okuda K., Sasaki H., Yukiue H., Yano M., Fujii Y. (2008). Met gene copy number predicts the prognosis for completely resected non-small cell lung cancer. Cancer Sci..

[10] Onozato R., Kosaka T., Kuwano H., Sekido Y., Yatabe Y., Mitsudomi T. (2009). Activation of MET by Gene Amplification or by Splice Mutations Deleting the Juxtamembrane Domain in Primary Resected Lung Cancers. J. Thorac. Oncol..

[11] Matsubara D., Ishikawa S., Sachiko O., Aburatani H., Fukayama M., Niki T. (2010). Co-Activation of Epidermal Growth Factor Receptor and c-MET Defines a Distinct Subset of Lung Adenocarcinomas. Am. J. Pathol..

[12] Ou S.-H.I., Kwak E.L., Siwak-Tapp C., Dy J., Bergethon K., Clark J.W., Camidge D.R., Solomon B.J., Maki R.G., Bang Y.-J. (2011). Activity of Crizotinib (PF02341066), a Dual Mesenchymal-Epithelial Transition (MET) and Anaplastic Lymphoma Kinase (ALK) Inhibitor, in a Non-small Cell Lung Cancer Patient with De Novo MET Amplification. J. Thorac. Oncol..

[13] Tsuta K., Kozu Y., Mimae T., Yoshida A., Kohno T., Sekine I., Tamura T., Asamura H., Furuta K., Tsuda H. (2012). c-MET/Phospho-MET Protein Expression and MET Gene Copy Number in Non-small Cell Lung Carcinomas. J. Thorac. Oncol..

[14] Park S., Choi Y.L., Sung C.O., An J., Seo J., Ahn M.J., Ahn J.S., Park K., Shin Y.K., Erkin O.C. (2012). High MET copy number and MET overexpression: Poor outcome in non-small cell lung cancer patients. Histol. Histopathol..

[15] Yu H.A., Arcila M.E., Rekhtman N., Sima C.S., Zakowski M.F., Pao W., Kris M.G., Miller V.A., Ladanyi M., Riely G.J. (2013). Analysis of Tumor Specimens at the Time of Acquired Resistance to EGFR-TKI Therapy in 155 Patients with EGFR-Mutant Lung Cancers. Clin. Cancer Res..

[16] Tong J.H., Yeung S.F., Chan A.W.H., Chung L.Y., Chau S.L., Lung R.W.M., Tong C.Y., Chow C., Tin E.K.Y., Yu Y.H. (2016). MET Amplification and Exon 14 Splice Site Mutation Define Unique Molecular Subgroups of Non–Small Cell Lung Carcinoma with Poor Prognosis. Clin. Cancer Res..

[17] Stransky N., Cerami E., Schalm S., Kim J.L., Lengauer C. (2014). The landscape of kinase fusions in cancer. Nat. Commun..

[18] Ma P.C. (2015). MET Receptor Juxtamembrane Exon 14 Alternative Spliced Variant: Novel Cancer Genomic Predictive Biomarker. Cancer Discov..

[19] Van Der Steen N., Giovannetti E., Pauwels P., Peters G.J., Hong D.S., Cappuzzo F., Hirsch F.R., Rolfo C. (2016). cMET Exon 14 Skipping: From the Structure to the Clinic. J. Thorac. Oncol..

[20] Heist R.S., Sequist L.V., Borger D., Gainor J.F., Arellano R.S., Le L.P., Dias-Santagata D., Clark J.W., Engelman J.A., Shaw A.T. (2016). Acquired Resistance to Crizotinib in NSCLC with MET Exon 14 Skipping. J. Thorac. Oncol..

[21] Ou S.-H.I., Young L., Schrock A.B., Johnson A., Klempner S.J., Zhu V.W., Miller V.A., Ali S.M. (2017). Emergence of Preexisting MET Y1230C Mutation as a Resistance Mechanism to Crizotinib in NSCLC with MET Exon 14 Skipping. J. Thorac. Oncol..

[22] Lee G.D., Lee S.E., Oh D.-Y., Yu D.-b., Jeong H.M., Kim J., Hong S., Jung H.S., Oh E., Song J.-Y. (2017). MET Exon 14 Skipping Mutations in Lung Adenocarcinoma: Clinicopathologic Implications and Prognostic Values. J. Thorac. Oncol..

[23] Shen M., Zhao X., Zhao L., Shi L., An S., Huang G., Liu J. (2018). Met is involved in TIGAR-regulated metastasis of non-small-cell lung cancer. Mol. Cancer.

[24] Saigi M., Alburquerque-Bejar J.J., Mc Leer-Florin A., Pereira C., Pros E., Romero O.A., Baixeras N., Esteve-Codina A., Nadal E., Brambilla E. (2018). MET-Oncogenic and JAK2-Inactivating Alterations Are Independent Factors That Affect Regulation of PD-L1 Expression in Lung Cancer. Clin. Cancer Res..

[25] Kim J.H., Kim H.S., Kim B.J. (2018). Prognostic value of MET copy number gain in non-small-cell lung cancer: An updated meta-analysis. J. Cancer.

[26] Ikeda S., Schwaederle M., Mohindra M., Fontes Jardim D.L., Kurzrock R. (2018). MET alterations detected in blood-derived circulating tumor DNA correlate with bone metastases and poor prognosis. J. Hematol. Oncol..

[27] Guo R., Berry L.D., Aisner D.L., Sheren J., Boyle T., Bunn P.A., Johnson B.E., Kwiatkowski D.J., Drilon A., Sholl L.M. (2019). MET IHC Is a Poor Screen for MET Amplification or MET Exon 14 Mutations in Lung Adenocarcinomas: Data from a Tri-Institutional Cohort of the Lung Cancer Mutation Consortium. J. Thorac. Oncol..

[28] Drilon A., Clark J.W., Weiss J., Ou S.-H.I., Camidge D.R., Solomon B.J., Otterson G.A., Villaruz L.C., Riely G.J., Heist R.S. (2020). Antitumor activity of crizotinib in lung cancers harboring a MET exon 14 alteration. Nat. Med..

[29] Mondelo-Macía P., Rodríguez-López C., Valiña L., Aguín S., León-Mateos L., García-González J., Abalo A., Rapado-González O., Suárez-Cunqueiro M., Díaz-Lagares A. (2020). Detection of MET Alterations Using Cell Free DNA and Circulating Tumor Cells from Cancer Patients. Cells.

[30] Jamme P., Fernandes M., Copin M.-C., Descarpentries C., Escande F., Morabito A., Grégoire V., Jamme M., Baldacci S., Tulasne D. (2020). Alterations in the PI3K Pathway Drive Resistance to MET Inhibitors in NSCLC Harboring MET Exon 14 Skipping Mutations. J. Thorac. Oncol..

[31] Cheng T., Gu Z., Song D., Liu S., Tong X., Wu X., Lin Z., Hong W. (2021). Genomic and clinical characteristics of MET exon14 alterations in a large cohort of Chinese cancer patients revealed distinct features and a novel resistance mechanism for crizotinib. J. Cancer.

[32] Guo R., Offin M., Brannon A.R., Chang J., Chow A., Delasos L., Girshman J., Wilkins O., McCarthy C.G., Makhnin A. (2021). MET Exon 14–altered Lung Cancers and MET Inhibitor Resistance. Clin. Cancer Res..

[33] Liu L., Kalyani F.S., Yang H., Zhou C., Xiong Y., Zhu S., Yang N., Qu J. (2021). Prognosis and Concurrent Genomic Alterations in Patients With Advanced NSCLC Harboring MET Amplification or MET Exon 14 Skipping Mutation Treated With MET Inhibitor: A Retrospective Study. Front. Oncol..

[34] Ahn M.-J., Mendoza M.J.L., Pavlakis N., Kato T., Soo R.A., Kim D.-W., Liam C.K., Hsia T.-C., Lee C.K., Reungwetwattana T. (2022). Asian Thoracic Oncology Research Group (ATORG) Expert Consensus Statement on MET Alterations in NSCLC: Diagnostic and Therapeutic Considerations. Clin. Lung Cancer.

[35] Tyler L.C., Le A.T., Chen N., Nijmeh H., Bao L., Wilson T.R., Chen D., Simmons B., Turner K.M., Perusse D. (2022). MET gene amplification is a mechanism of resistance to entrectinib in ROS1+ NSCLC. Thorac. Cancer.

[36] Qin K., Hong L., Zhang J., Le X. (2023). MET Amplification as a Resistance Driver to TKI Therapies in Lung Cancer: Clinical Challenges and Opportunities. Cancers.

[37] Yao Y., Yang H., Zhu B., Wang S., Pang J., Wu X., Xu Y., Zhang J., Zhang J., Ou Q. (2023). Mutations in the MET tyrosine kinase domain and resistance to tyrosine kinase inhibitors in non-small-cell lung cancer. Respir. Res..

[38] Sun D., Wu W., Wang L., Qu J., Han Q., Wang H., Song S., Liu N., Wang Y., Hou H. (2023). Identification of MET fusions as novel therapeutic targets sensitive to MET inhibitors in lung cancer. J. Transl. Med..

[39] Ouyang G., Shu P., Xue Y., Luo F., Li Y. (2024). Response to Savolitinib in a Patient with Advanced Poorly Differentiated Lung Carcinoma Positive for a Novel EML4-MET Gene Fusion. OncoTargets Ther..

[40] Alves de Souza G., Dornellas D.M.S., Campregher P.V., Teixeira C.H.A., Schvartsman G. (2024). Complete response to capmatinib in a patient with metastatic lung adenocarcinoma harboring CD47-MET fusion: A case report. Oncologist.

[41] Cheng W., Xu T., Yang L., Yan N., Yang J., Fang S. (2024). Dramatic response to crizotinib through MET phosphorylation inhibition in rare TFG-MET fusion advanced squamous cell lung cancer. Oncologist.

[42] Dias e Silva D., Mambetsariev I., Fricke J., Babikian R., Dingal S.T., Mazdisnian F., Badie B., Arvanitis L., Afkhami M., Villalona-Calero M. (2024). A novel HLA-DQB2::MET gene fusion variant in lung adenocarcinoma with prolonged response to tepotinib: A case report. Transl. Lung Cancer Res..

[43] Kontic M., Stjepanovic M., Markovic F. (2025). Beyond the Tissue: Unlocking NSCLC Treatment Potential Through Liquid Biopsy. Genes.

[44] Rolfo C., O’Brate A., Menzel C., Bruns R., Juraeva D., Stroh C., Johne A., Paik P.K. (2025). Liquid and Tissue Biopsies for Identifying MET Exon 14 Skipping NSCLC: Analyses from the Phase II VISION Study of Tepotinib. Clin. Cancer Res..

[45] Tanizaki J., Okamoto I., Okamoto K., Takezawa K., Kuwata K., Yamaguchi H., Nakagawa K. (2011). MET Tyrosine Kinase Inhibitor Crizotinib (PF-02341066) Shows Differential Antitumor Effects in Non-small Cell Lung Cancer According to MET Alterations. J. Thorac. Oncol..

[46] Sequist L.V., von Pawel J., Garmey E.G., Akerley W.L., Brugger W., Ferrari D., Chen Y., Costa D.B., Gerber D.E., Orlov S. (2011). Randomized Phase II Study of Erlotinib Plus Tivantinib Versus Erlotinib Plus Placebo in Previously Treated Non–Small-Cell Lung Cancer. J. Clin. Oncol..

[47] https://clinicaltrials.gov.

[48] Goździk-Spychalska J., Szyszka-Barth K., Spychalski Ł., Ramlau K., Wójtowicz J., Batura-Gabryel H., Ramlau R. (2014). c-MET Inhibitors in the Treatment of Lung Cancer. Curr. Treat. Options Oncol..

[49] Koeppen H., Rost S., Yauch R.L. (2013). Developing biomarkers to predict benefit from HGF/MET pathway inhibitors. J. Pathol..

[50] Ou S.-H.I., Govindan R., Eaton K.D., Otterson G.A., Gutierrez M.E., Mita A.C., Argiris A., Brega N.M., Usari T., Tan W. (2017). Phase I Results from a Study of Crizotinib in Combination with Erlotinib in Patients with Advanced Nonsquamous Non–Small Cell Lung Cancer. J. Thorac. Oncol..

[51] Drilon A., Clark J., Weiss J., Ou S., Camidge D.R., Solomon B., Otterson G., Villaruz L., Riely G., Heist R. (2018). OA12.02 Updated Antitumor Activity of Crizotinib in Patients with MET Exon 14-Altered Advanced Non-Small Cell Lung Cancer. J. Thorac. Oncol..

[52] Felip E., Sakai H., Patel J., Horn L., Veillon R., Griesinger F., Bruns R., Scheele J., Paik P. (2018). OA12.01 Phase II Data for the MET Inhibitor Tepotinib in Patients with Advanced NSCLC and MET Exon 14-Skipping Mutations. J. Thorac. Oncol..

[53] Wolf J., Seto T., Han J.-Y., Reguart N., Garon E.B., Groen H.J.M., Tan D.S.-W., Hida T., De Jonge M.J., Orlov S.V. (2019). Capmatinib (INC280) in METΔex14-mutated advanced non-small cell lung cancer (NSCLC): Efficacy data from the phase II GEOMETRY mono-1 study. J. Clin. Oncol..

[54] Paik P.K., Veillon R., Cortot A.B., Felip E., Sakai H., Mazieres J., Griesinger F., Horn L., Senellart H., Van Meerbeeck J.P. (2019). Phase II study of tepotinib in NSCLC patients with METex14 mutations. J. Clin. Oncol..

[55] Paik P.K., Felip E., Veillon R., Sakai H., Cortot A.B., Garassino M.C., Mazieres J., Viteri S., Senellart H., Van Meerbeeck J. (2020). Tepotinib in Non–Small-Cell Lung Cancer with MET Exon 14 Skipping Mutations. N. Engl. J. Med..

[56] Oxnard G.R., Yang J.C.H., Yu H., Kim S.W., Saka H., Horn L., Goto K., Ohe Y., Mann H., Thress K.S. (2020). TATTON: A multi-arm, phase Ib trial of osimertinib combined with selumetinib, savolitinib, or durvalumab in EGFR-mutant lung cancer. Ann. Oncol..

[57] Markham A. (2020). Tepotinib: First Approval. Drugs.

[58] Dhillon S. (2020). Capmatinib: First Approval. Drugs.

[59] Guo M.Z., Marrone K.A., Spira A., Waterhouse D.M., Scott S.C. (2021). Targeted Treatment of Non-Small Cell Lung Cancer: Focus on Capmatinib with Companion Diagnostics. OncoTargets Ther..

[60] Markham A. (2021). Savolitinib: First Approval. Drugs.

[61] Leighl N.B., Shu C.A., Minchom A., Felip E., Cousin S., Cho B.C., Park K., Han J.Y., Boyer M., Lee C.K. (2021). 1192MO Amivantamab monotherapy and in combination with lazertinib in post-osimertinib EGFR-mutant NSCLC: Analysis from the CHRYSALIS study. Ann. Oncol..

[62] Neijssen J., Cardoso R.M.F., Chevalier K.M., Wiegman L., Valerius T., Anderson G.M., Moores S.L., Schuurman J., Parren P.W.H.I., Strohl W.R. (2021). Discovery of amivantamab (JNJ-61186372), a bispecific antibody targeting EGFR and MET. J. Biol. Chem..

[63] Fujino T., Suda K., Koga T., Hamada A., Ohara S., Chiba M., Shimoji M., Takemoto T., Soh J., Mitsudomi T. (2022). Foretinib can overcome common on-target resistance mutations after capmatinib/tepotinib treatment in NSCLCs with MET exon 14 skipping mutation. J. Hematol. Oncol..

[64] Coleman N., Yap T.A., Heymach J.V., Meric-Bernstam F., Le X. (2023). Antibody-drug conjugates in lung cancer: Dawn of a new era?. npj Precis. Oncol..

[65] Veillon R., Sakai H., Le X., Felip E., Cortot A.B., Smit E.F., Park K., Griesinger F., Britschgi C., Wu Y.-L. (2022). Safety of Tepotinib in Patients With MET Exon 14 Skipping NSCLC and Recommendations for Management. Clin. Lung Cancer.

[66] Mazieres J., Paik P.K., Garassino M.C., Le X., Sakai H., Veillon R., Smit E.F., Cortot A.B., Raskin J., Viteri S. (2023). Tepotinib treatment in patients with MET exon 14–skipping non–small cell lung cancer: Long-term follow-up of the VISION phase 2 nonrandomized clinical trial. JAMA Oncol..

[67] US Food and Drug Administration (2021). FDA Approves Tepotinib for Metastatic Non-Small Cell Lung Cancer.

[68] Zhao C., Lu D., Gao J. (2025). Telisotuzumab vedotin: The first-in-class c-Met-targeted antibody-drug conjugate granted FDA accelerated approval for treatment of non-squamous non-small cell lung cancer (NSCLC). Drug Discov. Ther..

[69] Uehara Y., Minowa O., Mori C., Shiota K., Kuno J., Noda T., Kitamura N. (1995). Placental defect and embryonic lethality in mice lacking hepatocyte growth factor/scatter factor. Nature.

[70] Huh C.-G., Factor V.M., Sánchez A., Uchida K., Conner E.A., Thorgeirsson S.S. (2004). Hepatocyte growth factor/c-metsignaling pathway is required for efficient liver regeneration and repair. Proc. Natl. Acad. Sci. USA.

[71] Trusolino L., Bertotti A., Comoglio P.M. (2010). MET signalling: Principles and functions in development, organ regeneration and cancer. Nat. Rev. Mol. Cell Biol..

[72] Ponzetto C., Bardelli A., Zhen Z., Maina F., dalla Zonca P., Giordano S., Graziani A., Panayotou G., Comoglio P.M. (1994). A multifunctional docking site mediates signaling and transformation by the hepatocyte growth factor/scatter factor receptor family. Cell.

[73] Kong-Beltran M., Stamos J., Wickramasinghe D. (2004). The Sema domain of Met is necessary for receptor dimerization and activation. Cancer Cell.

[74] Comoglio P.M. (2001). Pathway specificity for Met signalling. Nat. Cell Biol..

[75] Weidner K.M., Di Cesare S., Sachs M., Brinkmann V., Behrens J., Birchmeier W. (1996). Interaction between Gab1 and the c-Met receptor tyrosine kinase is responsible for epithelial morphogenesis. Nature.

[76] Gherardi E., Birchmeier W., Birchmeier C., Woude G.V. (2012). Targeting MET in cancer: Rationale and progress. Nat. Rev. Cancer.

[77] Drilon A., Cappuzzo F., Ou S.-H.I., Camidge D.R. (2017). Targeting MET in Lung Cancer: Will Expectations Finally Be MET?. J. Thorac. Oncol..

[78] Lee J., Kim S.T., Park S., Lee S., Park S.H., Park J.O., Lim H.Y., Ahn H., Bok H., Kim K.-M. (2018). Phase I Trial of Anti-MET Monoclonal Antibody in MET-Overexpressed Refractory Cancer. Clin. Color. Cancer.

[79] Hong L., Zhang J., Heymach J.V., Le X. (2021). Current and future treatment options for MET exon 14 skipping alterations in non-small cell lung cancer. Ther. Adv. Med. Oncol..

[80] Friedlaender A., Drilon A., Banna G.L., Peters S., Addeo A. (2020). The METeoric rise of MET in lung cancer. Cancer.

[81] Organ S.L., Tsao M.-S. (2011). An overview of the c-MET signaling pathway. Ther. Adv. Med. Oncol..

[82] Comoglio P.M., Giordano S., Trusolino L. (2008). Drug development of MET inhibitors: Targeting oncogene addiction and expedience. Nat. Rev. Drug Discov..

[83] Rosell R., Jantus-Lewintre E., Cao P., Cai X., Xing B., Ito M., Gomez-Vazquez J.L., Marco-Jordán M., Calabuig-Fariñas S., Cardona A.F. (2024). KRAS-mutant non-small cell lung cancer (NSCLC) therapy based on tepotinib and omeprazole combination. Cell Commun. Signal..

[84] Park S., Cho E.A., Chun J.N., Lee D.Y., Lee S., Kim M.Y., Bae S.M., Jo S.I., Lee S.H., Park H.H. (2022). Crizotinib attenuates cancer metastasis by inhibiting TGFβ signaling in non-small cell lung cancer cells. Exp. Mol. Med..

[85] Liu X., Newton R.C., Scherle P.A. (2010). Developing c-MET pathway inhibitors for cancer therapy: Progress and challenges. Trends Mol. Med..

[86] Xiao G.-H., Jeffers M., Bellacosa A., Mitsuuchi Y., Vande Woude G.F., Testa J.R. (2000). Anti-apoptotic signaling by hepatocyte growth factor/Met via the phosphatidylinositol 3-kinase/Akt and mitogen-activated protein kinase pathways. Proc. Natl. Acad. Sci. USA.

[87] Owusu B.Y., Thomas S., Venukadasula P., Han Z., Janetka J.W., Galemmo R.A., Klampfer L. (2017). Targeting the tumor-promoting microenvironment in MET-amplified NSCLC cells with a novel inhibitor of pro-HGF activation. Oncotarget.

[88] Sattler M., Hasina R., Reddy M.M., Gangadhar T., Salgia R. (2011). The role of the c-Met pathway in lung cancer and the potential for targeted therapy. Ther. Adv. Med. Oncol..

[89] The Cancer Genome Atlas Research Network (2014). Comprehensive molecular profiling of lung adenocarcinoma. Nature.

[90] Frampton G.M., Ali S.M., Rosenzweig M., Chmielecki J., Lu X., Bauer T.M., Akimov M., Bufill J.A., Lee C., Jentz D. (2015). Activation of MET via Diverse Exon 14 Splicing Alterations Occurs in Multiple Tumor Types and Confers Clinical Sensitivity to MET Inhibitors. Cancer Discov..

[91] Awad M.M., Oxnard G.R., Jackman D.M., Savukoski D.O., Hall D., Shivdasani P., Heng J.C., Dahlberg S.E., Jänne P.A., Verma S. (2016). MET Exon 14 Mutations in Non–Small-Cell Lung Cancer Are Associated With Advanced Age and Stage-Dependent MET Genomic Amplification and c-Met Overexpression. J. Clin. Oncol..

[92] Salgia R. (2017). MET in Lung Cancer: Biomarker Selection Based on Scientific Rationale. Mol. Cancer Ther..

[93] Sadiq A.A., Salgia R. (2013). MET As a Possible Target for Non–Small-Cell Lung Cancer. J. Clin. Oncol..

[94] Smyth E., Sclafani F., Cunningham D. (2014). Emerging molecular targets in oncology: Clinical potential of MET/hepatocyte growth-factor inhibitors. OncoTargets Ther..

[95] Sabari J.K., Leonardi G.C., Shu C.A., Umeton R., Montecalvo J., Ni A., Chen R., Dienstag J., Mrad C., Bergagnini I. (2018). PD-L1 expression, tumor mutational burden, and response to immunotherapy in patients with MET exon 14 altered lung cancers. Ann. Oncol..

[96] Paik P.K., Iams W.T., Husain H., O’Hara R.M., Adewusi E., Le X. (2025). Tepotinib in patients with MET exon 14 skipping non-small cell lung cancer. Cancer Treat. Rev..

[97] Pecci F., Li H., Di Federico A., Wu J., Chen H., Gariazzo E., Mantuano F., Garbo E., Aldea M., Santo V. (2025). First-line MET tyrosine kinase inhibitors versus immunotherapy ± chemotherapy for patients with MET exon 14 skipping mutant metastatic NSCLC. Clin. Cancer Res..

[98] Kawakami H., Okamoto I., Okamoto W., Tanizaki J., Nakagawa K., Nishio K. (2014). Targeting MET Amplification as a New Oncogenic Driver. Cancers.

[99] Albertson D.G., Collins C., McCormick F., Gray J.W. (2003). Chromosome aberrations in solid tumors. Nat. Genet..

[100] Hellman A., Zlotorynski E., Scherer S.W., Cheung J., Vincent J.B., Smith D.I., Trakhtenbrot L., Kerem B. (2002). A role for common fragile site induction in amplification of human oncogenes. Cancer Cell.

[101] Schildhaus H.-U., Schultheis A.M., Rüschoff J., Binot E., Merkelbach-Bruse S., Fassunke J., Schulte W., Ko Y.-D., Schlesinger A., Bos M. (2015). MET Amplification Status in Therapy-Naïve Adeno- and Squamous Cell Carcinomas of the Lung. Clin. Cancer Res..

[102] Zhang J., Babic A. (2016). Regulation of the MET oncogene: Molecular mechanisms. Carcinogenesis.

[103] Hwang C.-I., Matoso A., Corney D.C., Flesken-Nikitin A., Körner S., Wang W., Boccaccio C., Thorgeirsson S.S., Comoglio P.M., Hermeking H. (2011). Wild-type p53 controls cell motility and invasion by dual regulation of MET expression. Proc. Natl. Acad. Sci. USA.

[104] Yoshioka H., Azuma K., Yamamoto N., Takahashi T., Nishio M., Katakami N., Ahn M.J., Hirashima T., Maemondo M., Kim S.W. (2015). A randomized, double-blind, placebo-controlled, phase III trial of erlotinib with or without a c-Met inhibitor tivantinib (ARQ 197) in Asian patients with previously treated stage IIIB/IV nonsquamous nonsmall-cell lung cancer harboring wild-type epidermal growth factor receptor (ATTENTION study). Ann. Oncol..

[105] Spigel D.R., Edelman M.J., O’Byrne K., Paz-Ares L., Mocci S., Phan S., Shames D.S., Smith D., Yu W., Paton V.E. (2017). Results From the Phase III Randomized Trial of Onartuzumab Plus Erlotinib Versus Erlotinib in Previously Treated Stage IIIB or IV Non–Small-Cell Lung Cancer: METLung. J. Clin. Oncol..

[106] Pelosi G., Gasparini P., Conte D., Fabbri A., Perrone F., Tamborini E., Pupa S.M., Ciravolo V., Caserini R., Rossi G. (2016). Synergistic Activation upon MET and ALK Coamplification Sustains Targeted Therapy in Sarcomatoid Carcinoma, a Deadly Subtype of Lung Cancer. J. Thorac. Oncol..

[107] Petronini P.G., Ortiz-Zapater E., Lee R.W., Owen W., Weitsman G., Fruhwirth G., Dunn R.G., Neat M.J., McCaughan F., Parker P. (2017). MET-EGFR dimerization in lung adenocarcinoma is dependent on EGFR mtations and altered by MET kinase inhibition. PLoS ONE.

[108] Noro R., Seike M., Zou F., Soeno C., Matsuda K., Sugano T., Nishijima N., Matsumoto M., Kitamura K., Kosaihira S. (2015). MET FISH-positive status predicts short progression-free survival and overall survival after gefitinib treatment in lung adenocarcinoma with EGFR mutation. BMC Cancer.

[109] Noonan S.A., Berry L., Lu X., Gao D., Barón A.E., Chesnut P., Sheren J., Aisner D.L., Merrick D., Doebele R.C. (2016). Identifying the Appropriate FISH Criteria for Defining MET Copy Number–Driven Lung Adenocarcinoma through Oncogene Overlap Analysis. J. Thorac. Oncol..

[110] Finocchiaro G., Toschi L., Gianoncelli L., Baretti M., Santoro A. (2015). Prognostic and predictive value of MET deregulation in non-small cell lung cancer. Ann. Transl. Med..

[111] Cappuzzo F., Marchetti A., Skokan M., Rossi E., Gajapathy S., Felicioni L., del Grammastro M., Sciarrotta M.G., Buttitta F., Incarbone M. (2009). Increased MET Gene Copy Number Negatively Affects Survival of Surgically Resected Non–Small-Cell Lung Cancer Patients. J. Clin. Oncol..

[112] Li J., Wang Y., Zhang B., Xu J., Cao S., Zhong H. (2020). Characteristics and response to crizotinib in lung cancer patients with MET amplification detected by next-generation sequencing. Lung Cancer.

[113] Yang H., Zhou Z., Lin L., Yang M., Li C., Li Z., Yu X., Lizaso A., Han-Zhang H., Li B. (2020). Characterization of MET exon 14 alteration and association with clinical outcomes of crizotinib in Chinese lung cancers. Lung Cancer.

[114] Ma P.C., Jagadeeswaran R., Jagadeesh S., Tretiakova M.S., Nallasura V., Fox E.A., Hansen M., Schaefer E., Naoki K., Lader A. (2005). Functional Expression and Mutations of c-Met and Its Therapeutic Inhibition with SU11274 and Small Interfering RNA in Non–Small Cell Lung Cancer. Cancer Res..

[115] Tsao M.-S., Liu N., Chen J.-R., Pappas J., Ho J., To C., Viallet J., Park M., Zhu H. (1998). Differential expression of Met/hepatocyte growth factor receptor in subtypes of non-small cell lung cancers. Lung Cancer.

[116] Wang Y., Chen Z., Han X., Li J., Guo H., Shi J. (2021). Acquired MET D1228N Mutations Mediate Crizotinib Resistance in Lung Adenocarcinoma with ROS1 Fusion: A Case Report. Oncologist.

[117] Qi J., McTigue M.A., Rogers A., Lifshits E., Christensen J.G., Jänne P.A., Engelman J.A. (2011). Multiple Mutations and Bypass Mechanisms Can Contribute to Development of Acquired Resistance to MET Inhibitors. Cancer Res..

[118] Gow C.-H., Liu Y.-N., Li H.-Y., Hsieh M.-S., Chang S.-H., Luo S.-C., Tsai T.-H., Chen P.-L., Tsai M.-F., Shih J.-Y. (2018). Oncogenic Function of a KIF5B-MET Fusion Variant in Non-Small Cell Lung Cancer. Neoplasia.

[119] Davies K.D., Ng T.L., Estrada-Bernal A., Le A.T., Ennever P.R., Camidge D.R., Doebele R.C., Aisner D.L. (2017). Dramatic Response to Crizotinib in a Patient With Lung Cancer Positive for an HLA-DRB1-MET Gene Fusion. JCO Precis. Oncol..

[120] Mino-Kenudson M. (2017). Immunohistochemistry for predictive biomarkers in non-small cell lung cancer. Transl. Lung Cancer Res..

[121] Chen T., Wang C., Cousens L., Chan T. (2008). Validation of IHC staining on phosphorylation of c-Met receptor in preclinical and clinical specimens of ARQ197 biomarker study. Cancer Res..

[122] Watermann I., Schmitt B., Stellmacher F., Müller J., Gaber R., Kugler C., Reinmuth N., Huber R.M., Thomas M., Zabel P. (2015). Improved diagnostics targeting c-MET in non-small cell lung cancer: Expression, amplification and activation?. Diagn. Pathol..

[123] Onitsuka T., Uramoto H., Ono K., Takenoyama M., Hanagiri T., Oyama T., Izumi H., Kohno K., Yasumoto K. (2010). Comprehensive Molecular Analyses of Lung Adenocarcinoma with Regard to the Epidermal Growth Factor Receptor, K-ras, MET, and Hepatocyte Growth Factor Status. J. Thorac. Oncol..

[124] Scott J.A., Lennerz J., Johnson M.L., Gordan L.N., Dumanois R.H., Quagliata L., Ritterhouse L.L., Cappuzzo F., Wang B., Xue M. (2024). Compromised Outcomes in Stage IV Non–Small-Cell Lung Cancer With Actionable Mutations Initially Treated Without Tyrosine Kinase Inhibitors: A Retrospective Analysis of Real-World Data. JCO Oncol. Pract..

[125] Zheng Q., Lin X., Qi W., Yin J., Li J., Wang Y., Wang W., Li W., Liang Z. (2024). NGS and FISH for MET amplification detection in EGFR TKI resistant non-small cell lung cancer (NSCLC) patients: A prospective, multicenter study in China. Lung Cancer.

[126] Du J., Wu X., Tong X., Wang X., Wei J., Yang Y., Chang Z., Mao Y., Shao Y.W., Liu B. (2017). Circulating tumor DNA profiling by next generation sequencing reveals heterogeneity of crizotinib resistance mechanisms in a gastric cancer patient with MET amplification. Oncotarget.

[127] Su J.-W., Weng C.-D., Lin X.-C., Fang M.-M., Xiao X., Zhang Y.-C., Zhang X.-C., Su J., Xu C.-R., Yan H.-H. (2024). Plasma ddPCR for the detection of MET amplification in advanced NSCLC patients: A comparative real-world study. Ther. Adv. Med. Oncol..

[128] Yang X., Li X., Yan J., Liu Y., Yin J., Shao W., Lu Y., Xue J. (2025). Response to EGFR/NTRK/MET Co-Inhibition Guided by Paired NGS in Advanced NSCLC With Acquired EGFR L858R/T790M/C797S Mutations. J. Natl. Compr. Cancer Netw..

[129] Scagliotti G., von Pawel J., Novello S., Ramlau R., Favaretto A., Barlesi F., Akerley W., Orlov S., Santoro A., Spigel D. (2015). Phase III Multinational, Randomized, Double-Blind, Placebo-Controlled Study of Tivantinib (ARQ 197) Plus Erlotinib Versus Erlotinib Alone in Previously Treated Patients With Locally Advanced or Metastatic Nonsquamous Non–Small-Cell Lung Cancer. J. Clin. Oncol..

[130] Catenacci D.V.T., Tebbutt N.C., Davidenko I., Murad A.M., Al-Batran S.-E., Ilson D.H., Tjulandin S., Gotovkin E., Karaszewska B., Bondarenko I. (2017). Rilotumumab plus epirubicin, cisplatin, and capecitabine as first-line therapy in advanced MET-positive gastric or gastro-oesophageal junction cancer (RILOMET-1): A randomised, double-blind, placebo-controlled, phase 3 trial. Lancet Oncol..

[131] Spigel D.R., Ervin T.J., Ramlau R.A., Daniel D.B., Goldschmidt J.H., Blumenschein G.R., Krzakowski M.J., Robinet G., Godbert B., Barlesi F. (2013). Randomized Phase II Trial of Onartuzumab in Combination With Erlotinib in Patients With Advanced Non–Small-Cell Lung Cancer. J. Clin. Oncol..

[132] Caparica R., Yen C.T., Coudry R., Ou S.-H.I., Varella-Garcia M., Camidge D.R., de Castro G. (2017). Responses to Crizotinib Can Occur in High-Level MET -Amplified Non–Small Cell Lung Cancer Independent of MET Exon 14 Alterations. J. Thorac. Oncol..

[133] Jorge S.E., Schulman S., Freed J.A., VanderLaan P.A., Rangachari D., Kobayashi S.S., Huberman M.S., Costa D.B. (2015). Responses to the multitargeted MET/ALK/ROS1 inhibitor crizotinib and co-occurring mutations in lung adenocarcinomas with MET amplification or MET exon 14 skipping mutation. Lung Cancer.

[134] Ettinger D.S., Aisner D.L., Wood D.E., Akerley W., Bauman J., Chang J.Y., Chirieac L.R., D’Amico T.A., Dilling T.J., Dobelbower M. (2018). NCCN Guidelines Insights: Non–Small Cell Lung Cancer, Version 5.2018. J. Natl. Compr. Cancer Netw..

[135] Mok T.S.K., Geater S.L., Su W.-C., Tan E.-H., Yang J.C.-H., Chang G.-C., Han M., Komarnitsky P., Payumo F., Garrus J.E. (2016). A Randomized Phase 2 Study Comparing the Combination of Ficlatuzumab and Gefitinib with Gefitinib Alone in Asian Patients with Advanced Stage Pulmonary Adenocarcinoma. J. Thorac. Oncol..

[136] Chin L.P., Soo R.A., Soong R., Ou S.-H.I. (2012). Targeting ROS1 with Anaplastic Lymphoma Kinase Inhibitors: A Promising Therapeutic Strategy for a Newly Defined Molecular Subset of Non–Small-Cell Lung Cancer. J. Thorac. Oncol..

[137] Lin J.J., Riely G.J., Shaw A.T. (2017). Targeting ALK: Precision Medicine Takes on Drug Resistance. Cancer Discov..

[138] Stone A. (2014). EGFR and c-Met Inhibitors are Effective in Reducing Tumorigenicity in Cancer. J. Carcinog. Mutagen..

[139] Broekman F. (2011). Tyrosine kinase inhibitors: Multi-targeted or single-targeted?. World J. Clin. Oncol..

[140] Reungwetwattana T., Liang Y., Zhu V., Ou S.-H.I. (2017). The race to target MET exon 14 skipping alterations in non-small cell lung cancer: The Why, the How, the Who, the Unknown, and the Inevitable. Lung Cancer.

[141] Ou S.-H.I., Azada M., Dy J., Stiber J.A. (2011). Asymptomatic Profound Sinus Bradycardia (Heart Rate ≤45) in Non-small Cell Lung Cancer Patients Treated with Crizotinib. J. Thorac. Oncol..

[142] Camidge D.R., Bang Y.-J., Kwak E.L., Iafrate A.J., Varella-Garcia M., Fox S.B., Riely G.J., Solomon B., Ou S.-H.I., Kim D.-W. (2012). Activity and safety of crizotinib in patients with ALK-positive non-small-cell lung cancer: Updated results from a phase 1 study. Lancet Oncol..

[143] Wu Y.-L., Zhang L., Kim D.-W., Liu X., Lee D.H., Yang J.C.-H., Ahn M.-J., Vansteenkiste J.F., Su W.-C., Felip E. (2018). Phase Ib/II Study of Capmatinib (INC280) Plus Gefitinib After Failure of Epidermal Growth Factor Receptor (EGFR) Inhibitor Therapy in Patients With EGFR-Mutated, MET Factor–Dysregulated Non–Small-Cell Lung Cancer. J. Clin. Oncol..

[144] Wolf J., Hochmair M., Han J.-Y., Reguart N., Souquet P.-J., Smit E.F., Orlov S.V., Vansteenkiste J., Nishio M., de Jonge M. (2024). Capmatinib in MET exon 14-mutated non-small-cell lung cancer: Final results from the open-label, phase 2 GEOMETRY mono-1 trial. Lancet Oncol..

[145] Ou L., Tang Y., Deng Y., Guo L., He Q., He T., Feng W. (2022). Case Report: Durable partial response to icotinib plus crizotinib in a lung adenocarcinoma patient with double uncommon EGFR G719D/L861Q mutations and an acquired novel CUX1-MET fusion. Front. Oncol..

[146] Zhu Y.-C., Wang W.-X., Song Z.-B., Zhang Q.-X., Xu C.-W., Chen G., Zhuang W., Lv T., Song Y. (2018). MET-UBE2H Fusion as a Novel Mechanism of Acquired EGFR Resistance in Lung Adenocarcinoma. J. Thorac. Oncol..

[147] Breindel J.L., Haskins J.W., Cowell E.P., Zhao M., Nguyen D.X., Stern D.F. (2013). EGF Receptor Activates MET through MAPK to Enhance Non–Small Cell Lung Carcinoma Invasion and Brain Metastasis. Cancer Res..

[148] Ko B., He T., Gadgeel S., Halmos B. (2017). MET/HGF pathway activation as a paradigm of resistance to targeted therapies. Ann. Transl. Med..

[149] Yamada T., Takeuchi S., Nakade J., Kita K., Nakagawa T., Nanjo S., Nakamura T., Matsumoto K., Soda M., Mano H. (2012). Paracrine Receptor Activation by Microenvironment Triggers Bypass Survival Signals and ALK Inhibitor Resistance in EML4-ALK Lung Cancer Cells. Clin. Cancer Res..

[150] Kogita A., Togashi Y., Hayashi H., Banno E.R.I., Terashima M., De Velasco M.A., Sakai K., Fujita Y., Tomida S., Takeyama Y. (2015). Activated MET acts as a salvage signal after treatment with alectinib, a selective ALK inhibitor, in ALK-positive non-small cell lung cancer. Int. J. Oncol..

[151] Tanimoto A., Yamada T., Nanjo S., Takeuchi S., Ebi H., Kita K., Matsumoto K., Yano S. (2014). Receptor ligand-triggered resistance to alectinib and its circumvention by Hsp90 inhibition in EML4-ALK lung cancer cells. Oncotarget.

[152] Bahcall M., Sim T., Paweletz C.P., Patel J.D., Alden R.S., Kuang Y., Sacher A.G., Kim N.D., Lydon C.A., Awad M.M. (2016). Acquired METD1228V Mutation and Resistance to MET Inhibition in Lung Cancer. Cancer Discov..

[153] Yano S., Wang W., Li Q., Matsumoto K., Sakurama H., Nakamura T., Ogino H., Kakiuchi S., Hanibuchi M., Nishioka Y. (2008). Hepatocyte Growth Factor Induces Gefitinib Resistance of Lung Adenocarcinoma with Epidermal Growth Factor Receptor–Activating Mutations. Cancer Res..

[154] Li A., Yang J.-j., Zhang X.-c., Zhang Z., Su J., Gou L.-y., Bai Y., Zhou Q., Yang Z., Han-Zhang H. (2017). Acquired MET Y1248H and D1246N Mutations Mediate Resistance to MET Inhibitors in Non–Small Cell Lung Cancer. Clin. Cancer Res..

[155] Scagliotti G.V., Shuster D., Orlov S., von Pawel J., Shepherd F.A., Ross J.S., Wang Q., Schwartz B., Akerley W. (2018). Tivantinib in Combination with Erlotinib versus Erlotinib Alone for EGFR-Mutant NSCLC: An Exploratory Analysis of the Phase 3 MARQUEE Study. J. Thorac. Oncol..

[156] Neal J.W., Dahlberg S.E., Wakelee H.A., Aisner S.C., Bowden M., Huang Y., Carbone D.P., Gerstner G.J., Lerner R.E., Rubin J.L. (2016). Erlotinib, cabozantinib, or erlotinib plus cabozantinib as second-line or third-line treatment of patients with EGFR wild-type advanced non-small-cell lung cancer (ECOG-ACRIN 1512): A randomised, controlled, open-label, multicentre, phase 2 trial. Lancet Oncol..

[157] Wakelee H.A., Gettinger S., Engelman J., Jänne P.A., West H., Subramaniam D.S., Leach J., Wax M., Yaron Y., Miles D.R. (2017). A phase Ib/II study of cabozantinib (XL184) with or without erlotinib in patients with non-small cell lung cancer. Cancer Chemother. Pharmacol..

[158] Reckamp K.L., Frankel P.H., Ruel N., Mack P.C., Gitlitz B.J., Li T., Koczywas M., Gadgeel S.M., Cristea M.C., Belani C.P. (2019). Phase II Trial of Cabozantinib Plus Erlotinib in Patients With Advanced Epidermal Growth Factor Receptor (EGFR)-Mutant Non-small Cell Lung Cancer With Progressive Disease on Epidermal Growth Factor Receptor Tyrosine Kinase Inhibitor Therapy: A California Cancer Consortium Phase II Trial (NCI 9303). Front. Oncol..

[159] Jänne P.A., Shaw A.T., Camidge D.R., Giaccone G., Shreeve S.M., Tang Y., Goldberg Z., Martini J.-F., Xu H., James L.P. (2016). Combined Pan-HER and ALK/ROS1/MET Inhibition with Dacomitinib and Crizotinib in Advanced Non–Small Cell Lung Cancer: Results of a Phase I Study. J. Thorac. Oncol..

[160] McCoach C.E., Yu A., Gandara D.R., Riess J.W., Vang D.P., Li T., Lara P.N., Gubens M., Lara F., Mack P.C. (2021). Phase I/II Study of Capmatinib Plus Erlotinib in Patients With MET-Positive Non–Small-Cell Lung Cancer. JCO Precis. Oncol..

[161] Pardo Aranda N., Remon J., Martinez Marti A., Martinez de Castro A.M., Cedres Perez S., Navarro A., Scheenaard E., Piera A., Carbonell L., Vivancos A. (2017). Outcome of EGFR mutant patirnts included in a clinical trial after progression on EGFR TKI. J. Clin. Oncol..

[162] Wu Y.-L., Cheng Y., Zhou J., Lu S., Zhang Y., Zhao J., Kim D.-W., Soo R.A., Kim S.-W., Pan H. (2020). Tepotinib plus gefitinib in patients with EGFR-mutant non-small-cell lung cancer with MET overexpression or MET amplification and acquired resistance to previous EGFR inhibitor (INSIGHT study): An open-label, phase 1b/2, multicentre, randomised trial. Lancet Respir. Med..

[163] Sequist L.V., Han J.-Y., Ahn M.-J., Cho B.C., Yu H., Kim S.-W., Yang J.C.-H., Lee J.S., Su W.-C., Kowalski D. (2020). Osimertinib plus savolitinib in patients with EGFR mutation-positive, MET-amplified, non-small-cell lung cancer after progression on EGFR tyrosine kinase inhibitors: Interim results from a multicentre, open-label, phase 1b study. Lancet Oncol..

[164] Shu C.A., Goto K., Ohe Y., Besse B., Park K., Wang Y., Griesinger F., Yang J.C.H., Felip E., Sanborn R.E. (2021). 1193MO Amivantamab plus lazertinib in post-osimertinib, post-platinum chemotherapy EGFR-mutant non-small cell lung cancer (NSCLC): Preliminary results from CHRYSALIS-2. Ann. Oncol..

[165] Zhao S., Ma Y., Liu L., Fang J., Ma H., Feng G., Xie B., Zeng S., Chang J., Ren J. (2024). Ningetinib plus gefitinib in EGFR-mutant non-small-cell lung cancer with MET and AXL dysregulations: A phase 1b clinical trial and biomarker analysis. Lung Cancer.

[166] Lutterbach B., Zeng Q., Davis L.J., Hatch H., Hang G., Kohl N.E., Gibbs J.B., Pan B.-S. (2007). Lung Cancer Cell Lines HarboringMETGene Amplification Are Dependent on Met for Growth and Survival. Cancer Res..

[167] Bean J., Brennan C., Shih J.-Y., Riely G., Viale A., Wang L., Chitale D., Motoi N., Szoke J., Broderick S. (2007). MET amplification occurs with or without T790M mutations in EGFR mutant lung tumors with acquired resistance to gefitinib or erlotinib. Proc. Natl. Acad. Sci. USA.

[168] Benedettini E., Sholl L.M., Peyton M., Reilly J., Ware C., Davis L., Vena N., Bailey D., Yeap B.Y., Fiorentino M. (2010). Met Activation in Non-Small Cell Lung Cancer Is Associated with de Novo Resistance to EGFR Inhibitors and the Development of Brain Metastasis. Am. J. Pathol..

[169] Solomon B. (2017). Trials and Tribulations of EGFR and MET Inhibitor Combination Therapy in NSCLC. J. Thorac. Oncol..

[170] Krebs M.G., Cho B.C., Hiret S., Han J.-Y., Lee K.H., Pérez C.L., De Braud F., Haura E.B., Sanborn R.E., Chih-Hsin Yang J. (2025). Amivantamab in Participants With Advanced NSCLC and MET Exon 14 Skipping Mutations: Final Results From the CHRYSALIS Study. J. Thorac. Oncol..

[171] Hartmaier R.J., Markovets A.A., Ahn M.J., Sequist L.V., Han J.-Y., Cho B.C., Yu H.A., Kim S.-W., Yang J.C.-H., Lee J.-S. (2023). Osimertinib + Savolitinib to Overcome Acquired MET-Mediated Resistance in Epidermal Growth Factor Receptor–Mutated, MET-Amplified Non–Small Cell Lung Cancer: TATTON. Cancer Discov..

[172] de Marinis F., Kim T.M., Bonanno L., Cheng S., Kim S.W., Tiseo M., Chu Q., Proto C., Sacher A., Luo Y.H. (2025). Savolitinib plus osimertinib in epidermal growth factor receptor (EGFR)-mutated advanced non-small cell lung cancer with MET overexpression and/or amplification following disease progression on osimertinib: Primary results from the phase II SAVANNAH study. Ann. Oncol..

[173] Shi P., Oh Y.-T., Zhang G., Yao W., Yue P., Li Y., Kanteti R., Riehm J., Salgia R., Owonikoko T.K. (2016). Met gene amplification and protein hyperactivation is a mechanism of resistance to both first and third generation EGFR inhibitors in lung cancer treatment. Cancer Lett..

[174] Zhang Y.-W., Staal B., Essenburg C., Su Y., Kang L., West R., Kaufman D., DeKoning T., Eagleson B., Buchanan S.G. (2010). MET Kinase Inhibitor SGX523 Synergizes with Epidermal Growth Factor Receptor Inhibitor Erlotinib in a Hepatocyte Growth Factor–Dependent Fashion to Suppress Carcinoma Growth. Cancer Res..

[175] Schrock A.B., Lai A., Ali S.M., Miller V.A., Raez L.E. (2017). Mutation of MET Y1230 as an Acquired Mechanism of Crizotinib Resistance in NSCLC with MET Exon 14 Skipping. J. Thorac. Oncol..

[176] Zhang W., Ai J., Shi D., Peng X., Ji Y., Liu J., Geng M., Li Y. (2014). Discovery of novel type II c-Met inhibitors based on BMS-777607. Eur. J. Med. Chem..

[177] McDermott U., Pusapati R.V., Christensen J.G., Gray N.S., Settleman J. (2010). Acquired Resistance of Non–Small Cell Lung Cancer Cells to MET Kinase Inhibition Is Mediated by a Switch to Epidermal Growth Factor Receptor Dependency. Cancer Res..

[178] Shin J.E., Park S., Jung H.A., Sun J.-M., Lee S.-H., Ahn J.S., Ahn M.-J. (2025). Combination of chemotherapy and immune checkpoint inhibitors in non-small cell lung cancer with actionable gene alterations other than EGFR, ALK, and ROS1 mutations: A retrospective observational study. BMC Cancer.

